# Proteomic analysis of cardiometabolic biomarkers and predictive modeling of severe outcomes in patients hospitalized with COVID-19

**DOI:** 10.1186/s12933-022-01569-7

**Published:** 2022-07-21

**Authors:** Philip H. Schroeder, Laura N. Brenner, Varinderpal Kaur, Sara J. Cromer, Katrina Armstrong, Regina C. LaRocque, Edward T. Ryan, James B. Meigs, Jose C. Florez, Richelle C. Charles, Josep M. Mercader, Aaron Leong

**Affiliations:** 1 Diabetes Unit, Endocrine Division, Department of Medicine, Massachusetts General Hospital, Boston, MA USA; 2 Center for Genomic Medicine, Massachusetts General Hospital, Boston, MA USA; 3 Programs in Metabolism and Medical and Population Genetics, Broad Institute of MIT and Harvard, Cambridge, MA USA; 4 Department of Medicine, Massachusetts General Hospital, Boston, MA USA; 5 Division of Pulmonary and Critical Care Medicine, Department of Medicine, Massachusetts General Hospital, Boston, MA USA; 6 Harvard Medical School, Boston, MA USA; 7 Division of Infectious Diseases, Department of Medicine, Massachusetts General Hospital, Boston, MA USA; 8 Department of Immunology and Infectious Diseases, Harvard T.H. Chan School of Public Health, Boston, MA USA; 9 Division of General Internal Medicine, Department of Medicine, Massachusetts General Hospital, 100 Cambridge St 16th Floor, Boston, MA 02114 USA

**Keywords:** COVID-19, Predictive modeling, Cardiometabolic biomarkers, Proteomics

## Abstract

**Background:**

The high heterogeneity in the symptoms and severity of COVID-19 makes it challenging to identify high-risk patients early in the disease. Cardiometabolic comorbidities have shown strong associations with COVID-19 severity in epidemiologic studies. Cardiometabolic protein biomarkers, therefore, may provide predictive insight regarding which patients are most susceptible to severe illness from COVID-19.

**Methods:**

In plasma samples collected from 343 patients hospitalized with COVID-19 during the first wave of the pandemic, we measured 92 circulating protein biomarkers previously implicated in cardiometabolic disease. We performed proteomic analysis and developed predictive models for severe outcomes. We then used these models to predict the outcomes of out-of-sample patients hospitalized with COVID-19 later in the surge (N = 194).

**Results:**

We identified a set of seven protein biomarkers predictive of admission to the intensive care unit and/or death (ICU/death) within 28 days of presentation to care. Two of the biomarkers, ADAMTS13 and VEGFD, were associated with a lower risk of ICU/death. The remaining biomarkers, ACE2, IL-1RA, IL6, KIM1, and CTSL1, were associated with higher risk. When used to predict the outcomes of the future, out-of-sample patients, the predictive models built with these protein biomarkers outperformed all models built from standard clinical data, including known COVID-19 risk factors.

**Conclusions:**

These findings suggest that proteomic profiling can inform the early clinical impression of a patient’s likelihood of developing severe COVID-19 outcomes and, ultimately, accelerate the recognition and treatment of high-risk patients.

**Supplementary Information:**

The online version contains supplementary material available at 10.1186/s12933-022-01569-7.

## Background

The COVID-19 pandemic, caused by the severe acute respiratory syndrome coronavirus 2 (SARS-CoV-2), has resulted in millions of deaths and persists as a global health threat [1]. COVID-19 causes a spectrum of symptoms, from mild upper respiratory tract infection to severe acute respiratory distress syndrome (ARDS) and death [2–4]. Most patients who develop severe COVID-19 deteriorate after one week of symptom onset [5]. As existing viral modifying therapies, such as monoclonal antibodies, are most effective when given before patients develop critical symptoms [6], early recognition is crucial to the triage and treatment of severe cases.

Previous studies of patients hospitalized with COVID-19 have consistently found significant associations between comorbidities and poor COVID-19 outcomes [7–29]. A cross-sectional study of over half a million hospitalized adults with COVID-19 found that 95% had at least one underlying medical condition and observed a strong dose–response association between the total number of underlying conditions (e.g., hypertension, obesity, lipid disorder, diabetes, and coronary artery disease) and risk of severe COVID-19 illness. Compared to patients with no documented underlying medical conditions, having one medical condition (7% of patients) was associated with 1.5 times higher risk of death, and having more than 10 conditions (17% of patients) was associated with a fourfold increase in the risk of death [7]. While little progress has been made in the development of tests that reliably predict which patients will suffer poor outcomes from COVID-19 (e.g., need for intensive care, assisted ventilation, septic shock, multiorgan system dysfunction, or death) [10], the presence of certain comorbid conditions, including higher body mass index (BMI) [7, 11–16], coronary artery disease [17–19], chronic kidney disease [20–25], and type 2 diabetes [7, 8, 21, 26–29], can help identify the patients most susceptible to severe forms of COVID-19.

Circulating protein biomarkers reflect underlying physiologic or disease states and can serve as measurable indicators of disease severity for prognostication in clinical practice. Interrogating the relationship between COVID-19 outcomes and protein biomarkers previously implicated in cardiometabolic disease may yield actionable insights regarding how common cardiometabolic comorbidities contribute to COVID-19 pathogenesis and, ultimately, improve our ability to predict which patients are most likely to suffer poor outcomes.

## Methods

### Study design

During the first wave of the pandemic (March 10 to June 1, 2020), we collected discarded blood samples from patients who presented to care (i.e., first contact with the healthcare system) with COVID-19 symptoms and were subsequently hospitalized with PCR-confirmed SARS-CoV-2 infection at Massachusetts General Hospital (MGH), a large academic hospital in Boston, Massachusetts. In these samples, we measured 92 protein biomarkers in the Olink Target 96 Cardiovascular II panel [30]. We performed proteomic analysis and developed predictive models for the combined outcome of death or admission to the intensive care unit (ICU/death) within 28 days of presentation to care in patients hospitalized early in the surge, between March 10 and April 21, 2020 (“in-sample” group; n = 343). We then used these models to predict the outcomes in a separate sample of patients hospitalized with COVID-19 later in the surge, between April 22 and June 1, 2020 (“out-of-sample” group; n = 194).

### Data collection

Details of the hospitalization, past medical history, and demographic characteristics were collected for each patient by manual chart reviews. Hospital laboratory tests performed within three days of presentation to care were retrieved electronically through the Enterprise Data Warehouse, a repository derived from electronic health records. We excluded hospital laboratory tests with a missing rate greater than 30% and imputed those remaining with the median of the non-missing values (mean missing rate = 13%). For each patient, we generated the CURB-65 score, a risk score for pneumonia severity [31]. The CURB-65 score ranges from 0 to 5 based on the presence of the following factors: (1) confusion (defined as Glasgow Coma Scale < 15), (2) blood urea nitrogen > 19 mg/dL, (3) respiratory rate ≥ 30, (4) systolic blood pressure < 90 mmHg or diastolic blood pressure ≤ 60 mmHg, and (5) age ≥ 65. Study procedures were approved by the Mass General Brigham (formerly Partners) Human Research Committee, the governing institutional review board at MGH.

### Plasma collection and proteomic assays

Blood samples were collected in EDTA tubes and processed within three days of collection in a biosafety level 2 + laboratory. Whole blood was centrifuged at 2800 rpm for 10 min. Plasma samples were extracted and stored at −80 °C. Prior to plating, samples were treated for two hours with 1% TritonX-100 at room temperature for virus inactivation. The Olink reagents were based on Proximity Extension Assay technology. Measurements were translated into normalized protein expression (NPX) units. Sample plates passed quality control if the standard deviations of internal controls were < 0.20 NPX and individual samples were < 0.30 NPX from the median value of the internal controls [30]. A total of 537 samples (95%) passed quality control. All 92 protein biomarkers were detected with intra-assay coefficient of variance (CV) < 15% (mean 4%) and inter-assay CV < 30% (mean 10%).

### Statistical analysis

We performed all statistical analyses in Python version 3.9.2 and R version 4.0.5. To account for non-normality in the raw data, we applied rank-based inverse normal transformation for feature selection, modeling, and *P* value calculation. To preserve interpretability, the odds ratios (ORs) shown in the volcano plot and correlation matrix were calculated after standardizing the data to have a mean of 0 and standard deviation of 1. To account for multiple testing, the threshold *P* < 0.05/116 hospital laboratory tests and protein biomarkers = 4 × 10^–4^ was used to determine significance.

To uncover mechanistic links, we performed a core enrichment analysis using QIAGEN Ingenuity Pathway Analysis (IPA; Ingenuity Systems, Redwood City, CA) by matching a proteomic dataset, consisting of *P* values and ORs for each protein biomarker, with curated content of the Ingenuity Knowledge Base. We generated reports for molecular networks and canonical pathways, highlighting upstream regulators and pathways relevant to the observed changes, along with their predicted impact on downstream biological or disease processes.

### Predictive modeling

To identify the subset of variables with the greatest predictive value for the logistic regression model, we ranked all variables by the frequency with which they were selected by LASSO (least absolute shrinkage and selection operator) regression when repeated across separate, random subsets of the in-sample data and different shrinkage parameters [32]. To identify the best model, we used an all-possible-regressions approach with these top-ranked variables. We used fivefold stratified cross-validation within the in-sample data to test the models formed by every possible combination of top-ranked variables. We then plotted the average area under the receiver operating characteristic curve (AUC), with the models sorted by the number of variables they included. The model that formed the elbow at which the plot of increasing AUC versus model size began to plateau was considered the best model, achieving the greatest performance without over-fitting the in-sample data. We performed this analysis with and without the 92 protein biomarkers and compared the AUCs of the best models built with and without the biomarkers using a two-sided DeLong test [33]. We repeated the modeling process using random forest models and using the Boruta algorithm to initially rank the variables by significance [34]. Data from the out-of-sample patients was not used at any point in the feature selection and modeling process.

## Results

### Characteristics of the patients

**Fig. 1:**
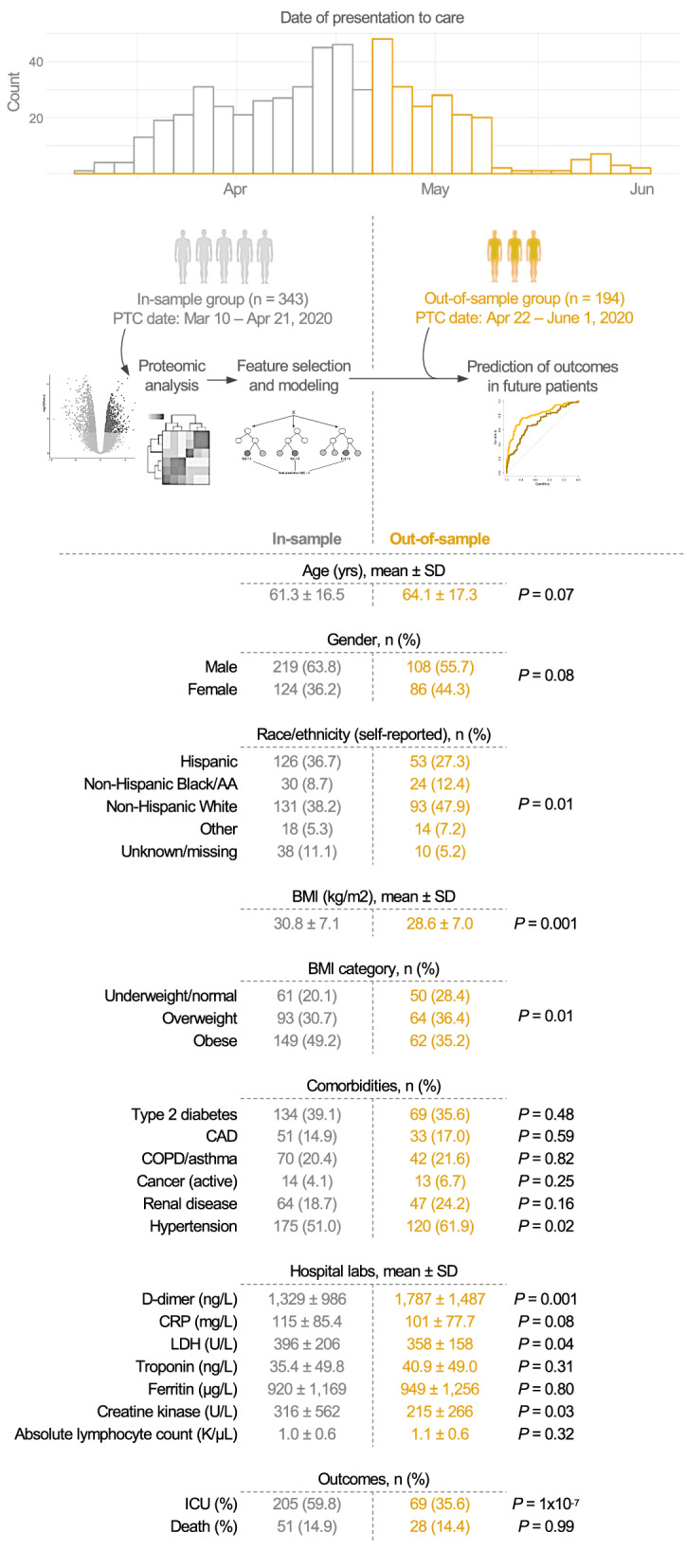
Characteristics of in-sample and out-of-sample patients. Blood samples were collected from 537 patients hospitalized with COVID-19 during an early surge in the outbreak, between March 10th and June 1st of 2020. Data from patients who were hospitalized early in the surge (before April 22, 2020) was used to analyze the cardiometabolic protein biomarkers and develop logistic regression and random forest models for severe outcomes. These patients comprised the in-sample group (shown in grey). These models were then used to predict the outcomes of the out-of-sample patients (shown in gold) who were hospitalized later in the surge (starting April 22, 2020). The in-sample and out-of-sample patients were compared across various demographic and clinical variables using a two-sided t-test for continuous variables and chi-square test for categorical variables. All race/ethnicity categories were self-reported. BMI categorization: < 18.5 kg/m^2^ for underweight, 18.5–24.9 kg/m^2^ for normal weight, 25.0–29.9 kg/m^2^ for overweight, and ≥ 30.0 kg/m^2^ for obese. *SD* Standard deviation, *AA* African American, *BMI* body mass index, *CAD* coronary artery disease, *COPD* chronic obstructive pulmonary disease, *CRP* C-reactive protein, *LDH* lactate dehydrogenase

Figure 1 shows the baseline characteristics of patients in the in-sample group and out-of-sample group. Table 1 shows the patient characteristics stratified by the ICU/death outcome in each group. Compared to the in-sample group, the out-of-sample group had a similar proportion of patients who died, but a smaller proportion of patients who were admitted to the ICU (Fig. 1). The out-of-sample group had a larger proportion of self-reported non-Hispanic White and non-Hispanic Black/African-American patients, a smaller proportion of self-reported Hispanic patients, a lower average BMI, higher levels of D-dimer, lower levels of lactate dehydrogenase, and lower levels of creatine kinase relative to the in-sample group. Patients who died or were admitted to the ICU had a higher average BMI, higher average CURB-65 score, and higher levels of D-dimer, LDH, C-reactive protein (CRP), and ferritin compared to those alive and not admitted to the ICU after 28 days of presentation to care (Table 1).

**Table 1:** Patient characteristics stratified by ICU/death outcome

	In-sample			Out-of-sample		
	No ICU/death	ICU/death		No ICU/death	ICU/death	
	(n=122)	(n=221)	*P*	(n=112)	(n=82)	*P*
Age (yrs), mean ± SD	62.2 ± 17.5	60.9 ± 16.0	0.47	64.3 ± 18.1	63.8 ± 16.3	0.83
Age (yrs), *n* (%)						
<65	68 (55.7)	132 (59.7)	0.55	53 (47.3)	39 (47.6)	1.0
≥65	54 (44.3)	89 (40.3)		59 (52.7)	43 (52.4)	
Gender,*n*(%)						
Male	75 (61.5)	144 (65.2)	0.57	56 (50.0)	52 (63.4)	0.09
Female	47 (38.5)	77 (34.8)		56 (50.0)	30 (36.6)	
Race/Ethnicity(Self-Reported),n(%)						
Hispanic	37 (30.0)	89 (40.3)	0.32	28 (25.0)	25 (30.5)	0.87
Non-HispanicBlack/AA	9 (7.4)	21 (9.5)		15 (13.4)	9 (11.0)	
Non-HispanicWhite	54 (44.3)	77 (34.8)		56 (50.0)	37 (45.1)	
Other	7 (5.7)	11 (5.0)		8 (7.1)	6 (7.3)	
Unknown/missing	15 (12.3)	23 (10.4)		5 (4.5)	5 (6.1)	
BMI(kg/m2),mean±SD	29.4±7.0	31.7±7.0	0.006	28±6.8	29.6±7.3	0.14
BMICategory,*n*(%)						
Underweight/normal	29 (25.7)	32 (16.8)	4×10^−4^	33 (31.1)	17 (24.3)	0.58
Overweight	45 (39.8)	48 (25.3)		38 (35.8)	26 (37.1)	
Obese	39 (34.5)	110 (57.9)		35 (33.0)	27 (38.6)	
CURB-65 Score, mean ± SD	1.0 ± 0.90	1.2 ± 1.0	0.003	1.3 ± 1.1	1.5 ± 1.1	0.20
Comorbidities, *n* (%)						
Type 2 diabetes	41 (33.6)	93 (42.1)	0.15	40 (35.7)	29 (35.4)	1.0
CAD	22 (18.0)	29 (13.1)	0.29	21 (18.8)	12 (14.6)	0.58
COPD/asthma	32 (26.2)	38 (17.2)	0.06	27 (24.1)	15 (18.3)	0.43
Cancer (active)	7 (5.7)	7 (3.2)	0.39	9 (8.0)	4 (4.9)	0.56
Renal disease	20 (16.4)	44 (19.9)	0.51	29 (25.9)	18 (22.0)	0.64
Hospital Labs, mean ± SD						
D-dimer (ng/L)	1006 ± 633	1510 ± 1,113	3×10^−5^	1,622 ± 1282	1971 ± 1674	0.14
CRP (mg/L)	72.6 ± 66.8	140 ± 85.9	5×10^−12^	82.1 ± 68.3	126 ± 80.2	2×10^−^^4^
LDH (U/L)	296 ± 97.9	452 ± 230	3×10^−11^	298 ± 94	435 ± 184	3×10^−9^
Troponin (ng/L)	31.8 ± 50.7	33.6 ± 37.9	0.76	42.3 ± 54.0	38.9 ± 40.8	0.69
Ferritin (µg/L)	526 ± 430	1118 ± 1360	1x10^−5^	672 ± 681	1502 ± 2052	2×10^−4^
Creatine kinase (U/L)	209 ± 335	376 ± 649	0.01	180 ± 238	250 ± 265	0.08
Absolute lymphocyte count (K/uL)	1.1 ± 0.5	1.0 ± 0.7	0.31	1.1 ± 0.6	0.9 ± 0.4	0.01

Patients who suffered the ICU/death outcome (defined as ICU admission or death within 28 days of presentation to care) were compared with those who did not suffer ICU/death across demographic factors, clinical variables, and hospital laboratory tests using a two-sided t-test for continuous variables and chi-square test for categorical variables. All race/ethnicity categories were self-reported. BMI categorization: < 18.5 kg/m^2^ for underweight, 18.5–24.9 kg/m^2^ for normal weight, 25.0–29.9 kg/m^2^ for overweight, and ≥ 30.0 kg/m^2^ for obese. *SD* Standard deviation, *AA* African American, *BMI* body mass index, *CAD* coronary artery disease, *COPD* chronic obstructive pulmonary disease, *CRP* C-reactive protein, *LDH* lactate dehydrogenase

### Cardiometabolic protein biomarkers

**Fig. 2:**
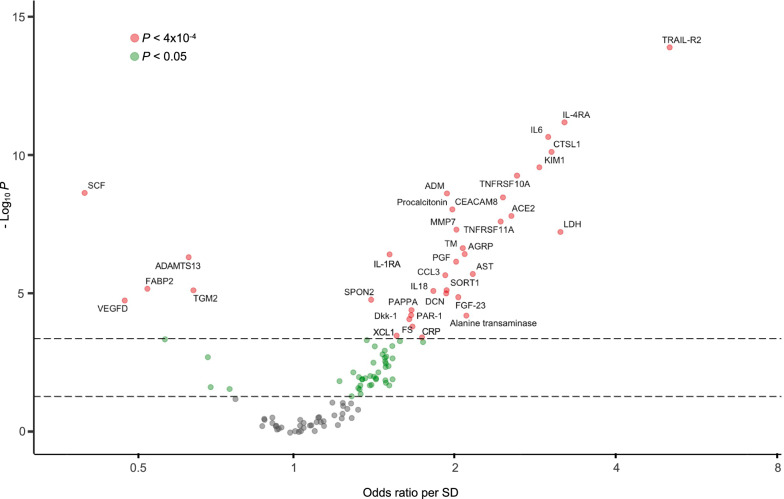
Volcano plot of the 92 cardiometabolic biomarkers and 24 hospital laboratory tests. The plot includes, for each protein biomarker and hospital lab, the odds ratio on the x-axis and *P* value (-log10) on the y-axis resulting from a logistic regression model with ICU/death as the outcome, adjusted for the covariates: age, gender, BMI, and self-reported race/ethnicity. To account for non-normality, the *P* values were calculated after applying rank-based inverse normal transformation. To preserve interpretability, the odds ratios were calculated from the data standardized to have a mean of 0 a standard deviation of 1. The threshold *P* < 0.05/116 hospital laboratory tests and protein biomarkers = 4 × 10^–4^ was used to identify significant results (shown in red). Nominally significant results (*P* < 0.05) are shown in green. *SD* Standard deviation, *CRP* C-reactive protein, *LDH* lactate dehydrogenase

To evaluate the relative importance of each of the 92 protein biomarkers and the 24 hospital laboratory tests with respect to ICU/death, we built a logistic regression model, adjusted for age, gender, BMI, and self-reported race/ethnicity, for each biomarker and hospital laboratory test in the in-sample group (Additional file 1: Table S1). The protein biomarkers comprised 31 of the 36 significant associations (*P* < 4 × 10^–4^) with ICU/death (Fig. 2). The five hospital laboratory tests with significant associations included LDH, CRP, procalcitonin, aspartate aminotransferase, and alanine transaminase, with standardized odds ratios (ORs) ranging from 1.8 to 3.2 (Additional file 1: Fig. S1).

**Fig. 3:**
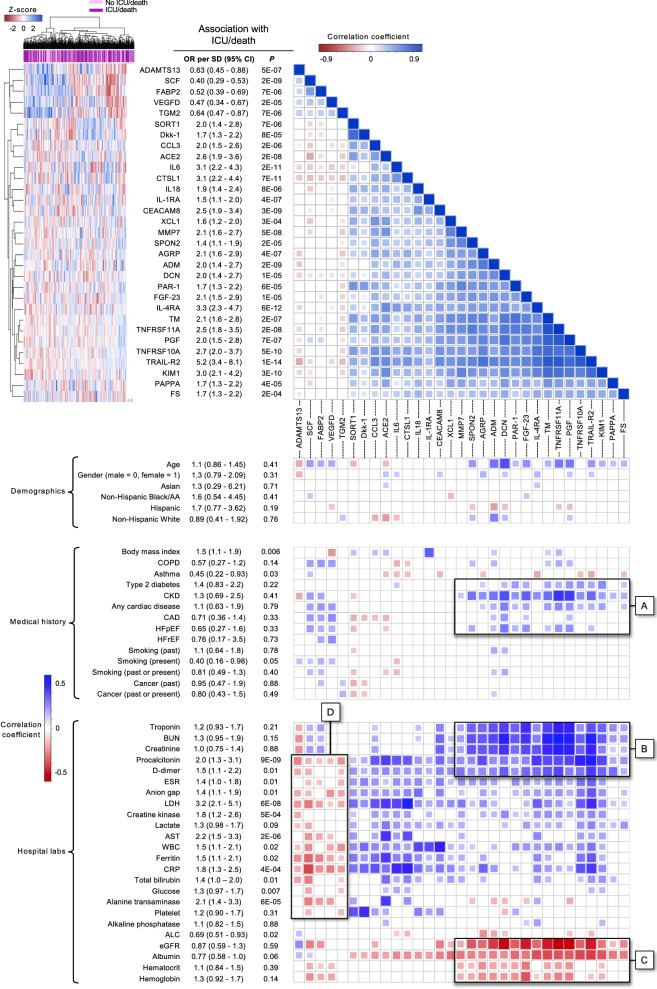
Hierarchical clustering and correlation matrix with significant cardiometabolic biomarkers. A heatmap (top left) and correlation matrix (top right and bottom) for the 31 protein biomarkers significantly associated with ICU/death (*P* < 0.05/116 hospital laboratory tests and biomarkers = 4 × 10^–4^). The correlation matrix shows how the protein biomarkers, ordered based on hierarchical clustering, correlate with one another (top right) and how they correlate with the demographic factors, clinical variables, and hospital laboratory tests (bottom). The color reflects the magnitude and direction of the Pearson correlation coefficient. The cells corresponding to correlations with *P* > 0.05 were left blank. The *P* values and odds ratios (OR) reported for the association of each variable with ICU/death are the same as those shown in Fig. 2. Box A shows the association of the largest cluster, comprised of 16 biomarkers, with type 2 diabetes, chronic kidney disease (CKD), and cardiac disease. Boxes B and C show how this cluster correlates with the hospital labs. Finally, Box D shows correlations between the hospital laboratory tests and a smaller cluster, comprising the five biomarkers that were negatively associated with ICU/death. *SD* Standard deviation, *CI* confidence interval, *AA* African American, *COPD* chronic obstructive pulmonary disease, *CAD* coronary artery disease, *HFpEF* heart failure with preserved ejection fraction, *HFrEF* heart failure with reduced ejection fraction, *BUN* blood urea nitrogen, *ERS* erythrocyte sedimentation rate, *LDH* lactate dehydrogenase, *AST* aspartate aminotransferase, *WBC* white blood cells, *CRP* C-reactive protein, *ALC* absolute lymphocyte count, *eGFR* estimated glomerular filtration rate

We evaluated how the protein biomarkers that were significantly associated with ICU/death correlated with one another and with the hospital laboratory tests, comorbidities, and demographics (Fig. 3). The largest cluster included 16 biomarkers which were positively associated with ICU/death (with standardized ORs ranging from 1.4 to 5.2) and showed positive correlations with type 2 diabetes, chronic kidney disease, and cardiac disease (Fig. 3: Box A). This cluster also showed significant positive correlations with troponin, blood urea nitrogen, creatinine, procalcitonin, and D-dimer (Fig. 3: Box B) and negative correlations with estimated glomerular filtration rate, albumin, hematocrit, and hemoglobin (Fig. 3: Box C). A smaller cluster of five biomarkers (ADAMTS13, SCF, FABP2, VEGFD, and TGM2) was negatively associated with ICU/death and negatively correlated with the majority of the hospital laboratory tests (Fig. 3: Box D). Among all 36 significant biomarkers and hospital laboratory tests, this cluster included the only markers associated with lower risk of ICU/death (with standardized ORs ranging from 0.40 to 0.64). Canonical pathways identified by IPA included the tumor microenvironment, IL-10 signaling, airway pathology, granulocyte adhesion, and wound healing. Implicated functional networks included cardiovascular and organismal development, lipid metabolism, and protein synthesis (Additional file 1: Tables S2 and S3).

### Prediction in out-of-sample patients

**Fig. 4:**
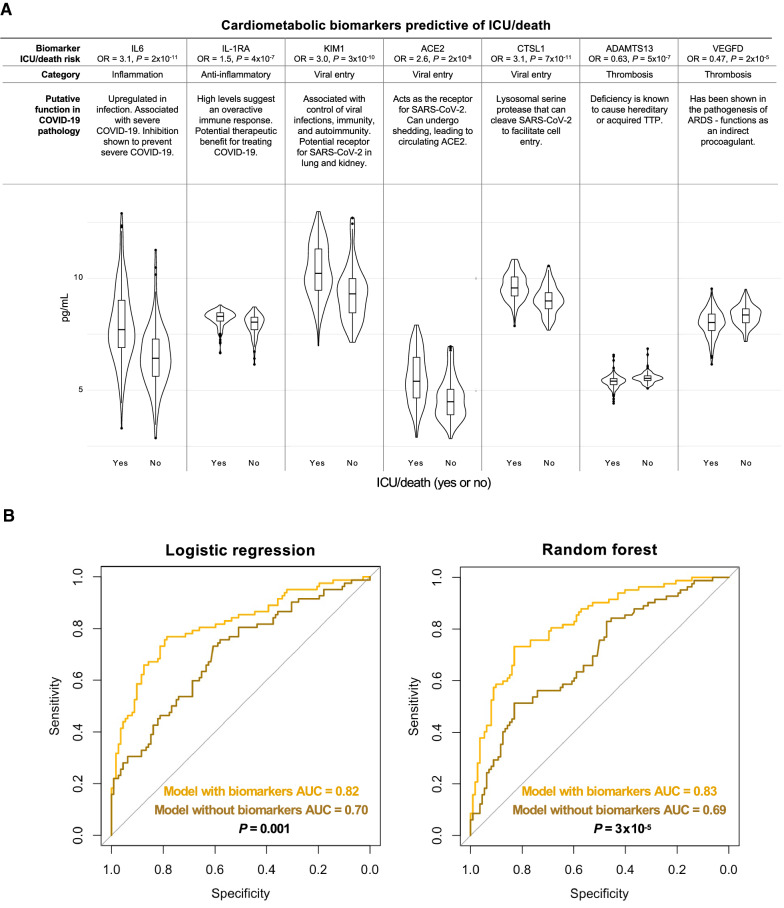
Prediction of ICU/death outcome in out-of-sample patients. **A** Violin plots for the set of seven cardiometabolic protein biomarkers that were included in the best model with biomarkers for both logistic regression and random forest. The figure depicts the distribution and box plot of these seven biomarkers, stratified by the ICU/death outcome, in the in-sample patient population. The *P* values shown for each biomarker are based on the rank-inverse normalized data, while the odds ratios (OR) are based on the data standardized to have a mean of 0 and standard deviation of 1. **B** The predictive performance of the best models with and without biomarkers in the out-of-sample patients. The figure shows the receiver operating characteristic curve and corresponding area under the curve (AUC) for the best logistic regression (left) and random forest (right) models with biomarkers (gold) and without biomarkers (bronze) in the out-of-sample patients. The best model with biomarkers, for both the logistic regression and random forest, included the same set of seven biomarker, shown in (A), along with two hospital labs: procalcitonin and LDH. All models were developed and trained using only the in-sample data. Thrombotic thrombocytopenic purpura, TTP; acute respiratory distress syndrome, ARDS

For both the logistic regression and random forest, the models built with the protein biomarkers outperformed the models built without the biomarkers in the out-of-sample patients (Fig. 4). The best-performing models consisted of a common set of nine variables (Table S4), which included two hospital laboratory measurements, procalcitonin and LDH (both of which were included in the best models without the biomarkers), and seven biomarkers: IL-1RA, CTSL1, ADAMTS13, VEGFD, KIM1, ACE2, and IL6 (Fig. 4A). The AUCs of the best models built with these nine variables were greater than that of the best models built without the protein biomarkers (logistic regression: 0.82 versus 0.70; *P* = 0.001; random forest: 0.83 versus 0.69; *P* = 3 × 10^–5^). We continued to observe superior performance by the models built with the protein biomarkers when excluding patients with more than 14 days between presentation to care and sample collection date (Additional file 1: Fig. S2 and Table S5), when excluding patients with sample collection on or after the ICU admission date (Additional file 1: Fig. S3), when randomly splitting patients into the in-sample and out-of-sample group (Additional file 1: Fig. S4), when stratifying patients by CURB-65 score and adjusting the models by CURB-65 score (Additional file 1: Table S6), and when directly adjusting each model by variables such as age, sex, and relevant comorbidities (e.g., BMI, COPD, asthma, type 2 diabetes, CKD, cardiac disease, smoking history, cancer history). Despite the loss of power, we see a similar difference in AUC between the model without biomarkers and the model with biomarkers (0.70 versus 0.79, p = 0.06) when excluding patients with a sample collection date greater than three days following presentation to care. The models with the protein biomarkers also outperformed the models without biomarkers in age-stratified and gender-stratified analyses (Additional file 1: Fig. S5 and S6). When evaluating the model performance in the two most prevalent self-reported categories of race/ethnicity, the improvement in performance of the model with biomarkers was greater in the Hispanic population than the non-Hispanic White population (Additional file 1: Fig. S7). We repeated the analysis using ICU admission as the outcome and death as the outcome. The results for the ICU admission outcome were similar to those for the combined ICU/death outcome (Fig. S8 and Table S7), while the results for the death outcome showed no significant difference in performance between the models built with and without the biomarkers (Additional file 1: Fig. S9 and Table S8).

## Discussion

We identified protein biomarkers previously implicated in cardiometabolic disease that were significantly associated with severe illness from COVID-19, shedding light on biological pathways involved in COVID-19 pathology. We demonstrated that these protein biomarkers, measured early in the disease course, were more predictive of ICU admissions or death than established clinical risk factors. These findings suggest that proteomic profiling could improve the triage and treatment of patients hospitalized with COVID-19.

We found a set of seven protein biomarkers (IL6, IL-1RA, KIM1, ACE2, CTSL1, ADAMTS13, and VEGFD), along with two hospital laboratory tests (procalcitonin and LDH), that were predictive of ICU/death. These circulating biomarkers are likely related to host and viral factors influencing disease, including inflammation (IL6, IL-1RA) [35, 36], thrombosis (ADAMTS13, VEGFD) [37, 38], and viral entry (KIM1, ACE2, CTSL1) [39–41]. Elevated inflammatory markers, including IL-6 [35, 42–44], CRP, ferritin, and D-dimer have been reported in severe COVID-19 [45]. Dexamethasone, an anti-inflammatory medication, and IL-6 receptor antagonists are current COVID-19 therapies shown to reduce the risk of poor outcomes in critically ill patients [46, 47].

LDH, D-dimer, fibrinogen, CRP, and low platelets, markers of thrombotic risk, have been reported to be associated with poor prognosis in COVID-19 [48]. This observation is in keeping with the association between lower ADAMTS13, an enzyme that degrades von Willebrand factor, and poor outcomes found in our study and other reports [37]. Low levels of ADAMTS13 have also been described in thrombotic thrombocytopenic purpura and syndromes of thrombotic microangiopathy caused by infection [49]. Microangiopathic thrombosis has been seen in autopsies of patients who have died of COVID-19, similar to what has been observed in other ARDS-causing diseases [50].

Three of the identified biomarkers, KIM1, ACE2, and CTSL, are involved in host-virus interactions. KIM1, an indicator of renal insults, plays a role in viral entry and regulation of the host immune response to viral infections [51]. ACE2, the cellular receptor for SARS-CoV-2 [40, 42], undergoes shedding, leading to circulating ACE2, a biomarker of cardiovascular disease, diabetes, and death in patients with and without COVID-19 [52, 53]. The association of ACE2 with severity is supported by a recently reported rare genetic variant that is associated with a 37% reduction in ACE2 expression and a 40% reduction in risk of severe COVID-19 [54]. Finally, CTSL is one of the lysosomal proteases that can cleave the SARS-CoV-2 spike protein, a step necessary for cellular entry [41, 55].

Previous studies have reported hospital laboratory tests and clinical characteristics associated with severe COVID-19 [5, 35, 36, 44, 45, 56–62]. These studies used both prospective and retrospective analyses of clinical variables, imaging findings, and laboratory values predictive of severity. Laboratory values such as IL6 [35, 44], IL-1RA, IL10 [36], D-dimer [5, 45, 61], and troponin [5, 60, 62] were associated with disease severity. Clinical characteristics, such as BMI, age [5, 63], history of renal failure, cardiovascular and cerebrovascular disease [60, 63], were also associated with worse outcomes. Our study shows the best predictive model included many of these previously reported risk factors when the protein biomarkers were not included (Table S4). However, when the protein biomarkers were included in the model-building process, these risk factors were replaced with the set of seven biomarkers, resulting in models that significantly outperformed all models developed from the clinical features and laboratory tests alone, suggesting that the biomarkers provide unique predictive value not captured by patient data and hospital lab values. The protein biomarkers replaced known clinical risk factors for severe illness that had been selected in the model built without biomarkers (i.e., BMI, D-dimer, CRP, ALC, and troponin). Notably, BMI was replaced by IL-1RA, a biomarker that was strongly correlated with BMI (Fig. 3). IL-1RA, known to be highly expressed in white adipose tissue [64] and upregulated during inflammation, could serve as a better proxy than BMI for obesity-driven COVID-19 risk. D-dimer was another previously reported COVID-19 risk factor [5, 45, 61] that was selected in the model without protein biomarkers but replaced in the model with biomarkers. D-dimer was positively correlated with IL6, IL-1RA, KIM1, ACE2, and CTSL1 (Fig. 3: Box B) and negatively correlated with ADAMTS13 and VEGFD (Fig. 3: Box D). The replacement of D-dimer in the final model with these seven protein biomarkers suggests that the predictive value provided by D-dimer was captured by this combination of biomarkers. Nevertheless, the utility of these protein biomarkers for the purpose of risk stratification in real-world clinical settings will need to be prospectively assessed in the context of their practical considerations (e.g., cost, speed of results reporting, and access to testing) for them to be appropriately incorporated in the clinical evaluation of patients hospitalized with COVID-19.

We recognize that standards of care and resource availability evolved quickly during the first wave of the pandemic. As data on the efficacy and side effects of COVID-19 therapies accrued, the use of remdesivir and dexamethasone increased, while the use of hydroxychloroquine decreased. MGH hospital guidelines did not recommend routine systemic anticoagulation for patients with COVID-19 during the pandemic and did not recommend the use of steroids, such as dexamethasone, until the publication of the RECOVERY study [65], after the recruitment period of this study. Prone positioning, applied heterogeneously early in the pandemic, eventually became standard of care. It is possible that these exogenous factors contributed to differences in outcomes between the in-sample and out-of-sample cohorts. We expect that, as the SARS-CoV-2 virus mutates, the virulence pathways and host responses may change, as noted by both the delta and omicron variants [66]. The patients hospitalized with COVID-19 today are generally younger and consist of both unvaccinated and vaccinated patients with breakthrough infections or repeat infections. Emerging COVID-19 therapies and medication exposures for preexisting medical conditions could influence the proteomic profile of patients and its association with COVID-19 severity.

By evaluating the models in a sample separate from that used to develop the models, we showed that the predictive value of the biomarkers was robust to changes in clinical protocols and the patient characteristics during the highly dynamic study period. Compared to studies that develop and test predictive models within the same patient population, our approach provided a more rigorous assessment of the generalizability of our models and the conclusions derived from our analysis. As one of the largest proteomic analyses performed in COVID-19 patients, we were able to conduct age-stratified, gender-stratified, and race/ethnicity-stratified analyses, demonstrating the strong performance of the model with the protein biomarkers across various demographic strata (Additional file 1: Fig. S5, S6, and S7).

Similar to previous COVID-19 analyses [67–69], this study was limited by the precision with which COVID-19 severity could be captured and COVID-19 related outcomes could be tracked. We used ICU admission and death as proxies for severe illness from COVID-19; however, patients may have died or been admitted to the ICU for reasons independent of their COVID-19 status. Further, patients who died after the 28-day follow-up period or outside the hospital or at other hospitals would not have been captured in our study. This underestimate of the true case-count for the death outcome may have biased results towards the null (Additional file 1: Fig. S9). Another limitation was that the hospital laboratory tests and protein biomarkers were not measured at the same time for all patients and the time between symptom onset and blood sample collection was not uniform across all patients. Despite this, we observed similar results when excluding patients with sample collection dates that were on or after the date of ICU admission or greater than three days or 14 days following presentation to care (Additional file 1: Fig. S2, Fig. S3, and Table S5). Finally, by only collecting discarded blood samples at a single time point, we were unable to perform longitudinal analyses; however, biomarkers that can be interpreted with single timepoint measurements may be more useful in clinical settings where only one lab draw is available. Our retrospective proteomic analysis shows that protein biomarkers improve prediction of severe outcomes over clinical biomarkers and risk factors that are routinely measured or obtained from hospitalized patients with COVID-19.

## Conclusion

In this study, we identified a set of protein biomarkers that yield both mechanistic insight regarding how cardiometabolic disease contributes to COVID-19 pathology, as well as predictive value regarding which patients have the highest risk for severe outcomes. If considered early in the clinical evaluation of patients with COVID-19, these insights can help clinicians estimate a patient’s cardiometabolic-driven risk, which, in turn, can inform downstream decisions regarding how to stratify patients across pathways of clinical care (e.g., in-hospital observation or early admission to ICU) and whether to institute treatments that reduce the risk of poor outcomes, such as monoclonal antibodies or novel antiviral therapies.

## Supplementary Information

**Additional file 1.** Additional figures and tables.

## Data Availability

The datasets generated and analyzed during the current study are not publicly available but may be made available upon reasonable request.

## Abbreviations

AA: African American
ACE2: Angiotensin converting enzyme 2
ADAMTS13: A disintegrin and metalloproteinase with a thrombospondin type 1 motif, member 13
ALC: Absolute lymphocyte count
ARDS: Acute respiratory distress syndrome
AST: Aspartate aminotransferase
AUC: Area under the curve
BMI: Body mass index
BUN: Blood urea nitrogen
CAD: Coronary artery disease
CKD: Chronic kidney disease
COPD: Chronic obstructive pulmonary disease
COVID-19: Coronavirus disease 19
CRP: C-reactive protein
CTSL1: Cathepsin L1
CV: Coefficient of variance
EGFR: Estimated glomerular filtration rate
ERS: Eerythrocyte sedimentation rate
HFpEF: Heart failure with preserved ejection fraction
HFrEF: Heart failure with reduced ejection fraction
ICU: Intensive care unit
IL-1RA: Interleukin 1 receptor antagonist
IL6: Interleukin 6
IPA: Ingenuity Pathway Analysis
KIM1: Kidney injury molecule 1
LASSO: Least absolute shrinkage and selection operator
LDH: Lactate dehydrogenase
MGH: Massachusetts General Hospital
NPX: Normalized protein expression
OR: Odds ratio
SARS-CoV-2: Severe acute respiratory syndrome coronavirus 2
SD: Standard deviation
TTP: Thrombotic thrombocytopenic purpura
VEGFD: Vascular endothelial growth factor D
WBC: White blood cells
