# Supplementary material for: Proteomic analysis of cardiometabolic biomarkers and predictive modeling of severe outcomes in patients hospitalized with COVID-19
Source: Cardiovasc Diabetol. 2022 Jul 21;21:136. doi: 10.1186/s12933-022-01569-7 (PMC9301894; doi:10.1186/s12933-022-01569-7)
Supplement: Supplementary file 1 — Additional file 1. Additional figures and tables. [file 12933_2022_1569_MOESM1_ESM.pdf]

S1 Fig. Violin plots of hospital labs significantly associated with ICU/death.

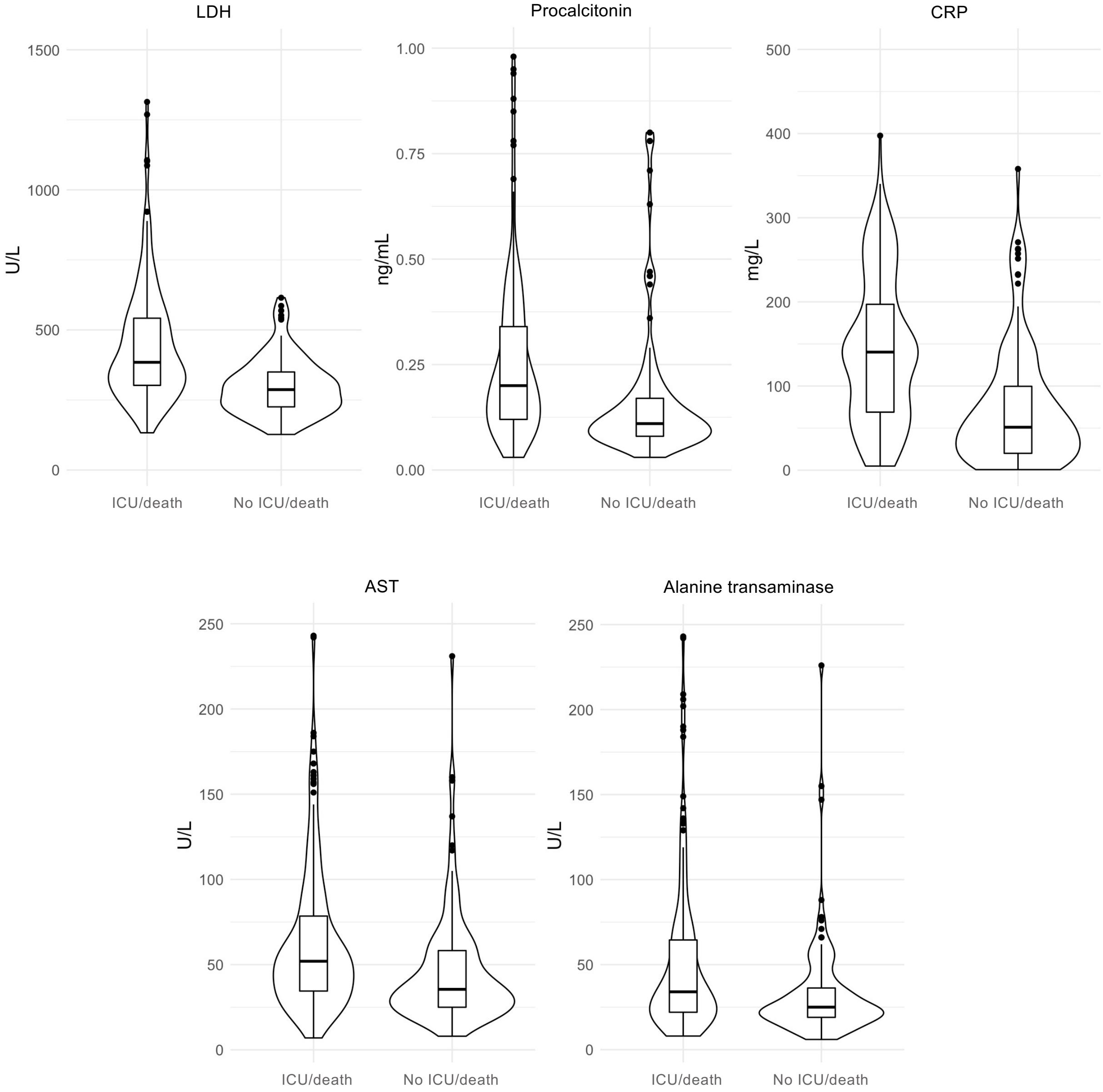

The plots include the distribution and box plots of the raw measurements, stratified by the ICU/death outcome, for the five hospital labs that were significantly associated ($P < 4{\times}10^{-4}$) with ICU/death. Lactate dehydrogenase, LDH; C-reactive protein, CRP; aspartate aminotransferase, AST.

S2 Fig. Prediction of ICU/death in out-of-sample patients with blood sample collection within 14 days of presentation to care.

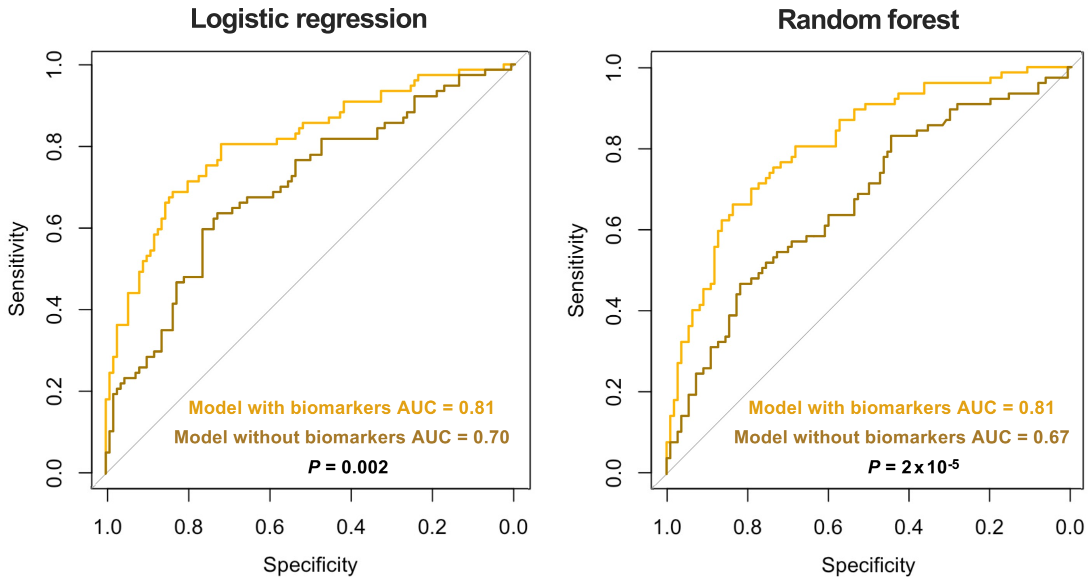

The figure shows the receiver operating characteristic curve and corresponding area under the curve (AUC) for the best logistic regression (left) and random forest (right) models for predicting ICU/death with biomarkers (gold) and without biomarkers (bronze) in the out-of-sample patients who had their blood sample collected less than 14 days (mean 5 days) after their presentation to care date.

S3 Fig. Prediction of ICU/death in out-of-sample patients with blood sample collection before ICU admission.

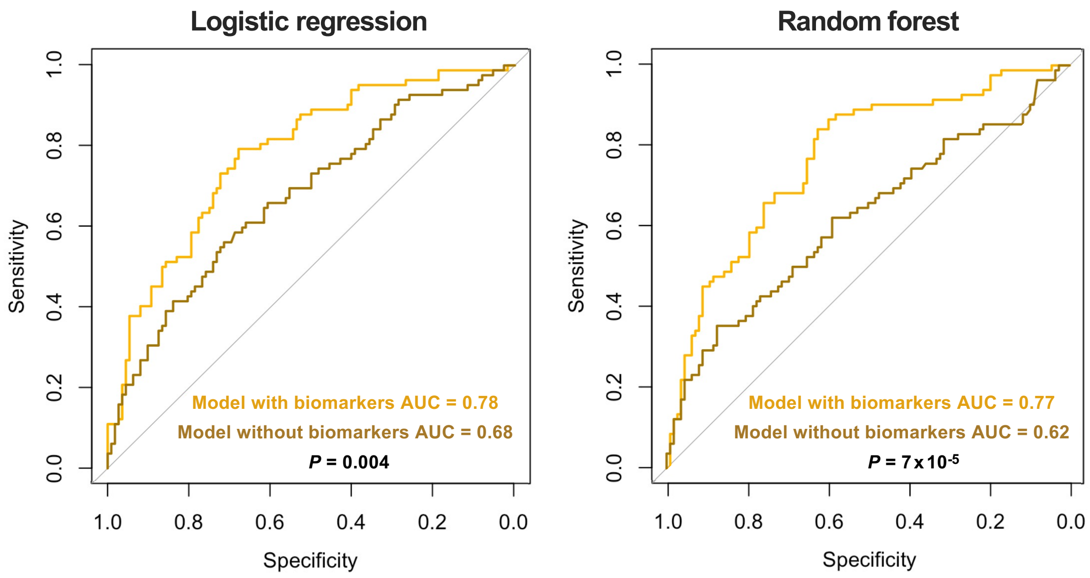

The figure shows the receiver operating characteristic curve and corresponding area under the curve (AUC) for the best logistic regression (left) and random forest (right) models for predicting ICU/death with biomarkers (gold) and without biomarkers (bronze) in the out-of-sample patients who had their blood sample collected prior to ICU admission.

S4 Fig. Prediction of ICU/death in out-of-sample patients with random split.

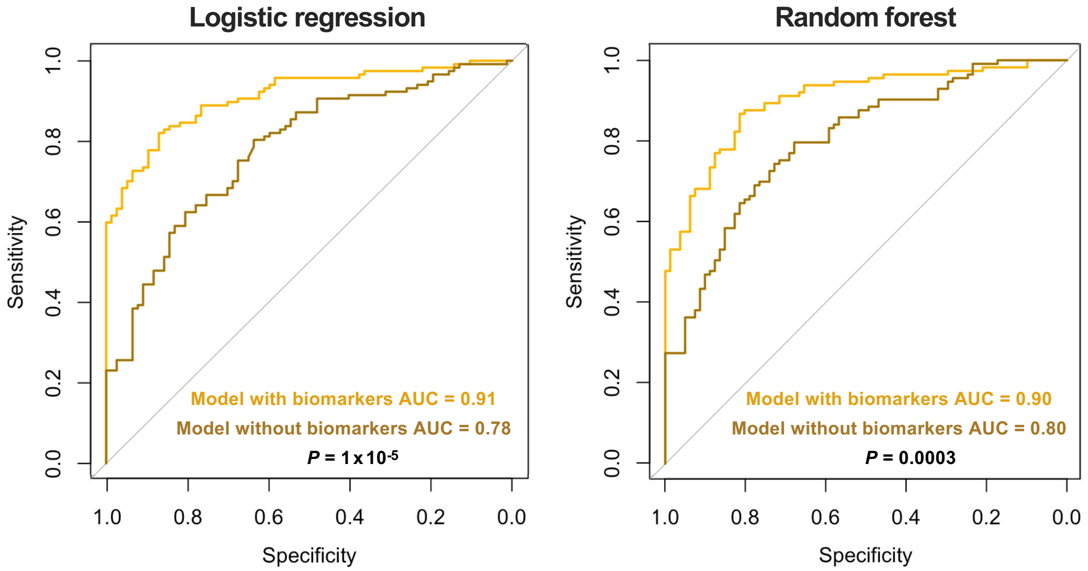

The figure shows the receiver operating characteristic curve and corresponding area under the curve (AUC) for the best logistic regression (left) and random forest (right) models for predicting ICU/death with biomarkers (gold) and without biomarkers (bronze) in the out-of-sample patients when using a random 50/50 split to group patients as in-sample and out-of-sample.

S5 Fig. Prediction of ICU/death in out-of-sample patients stratified by age.

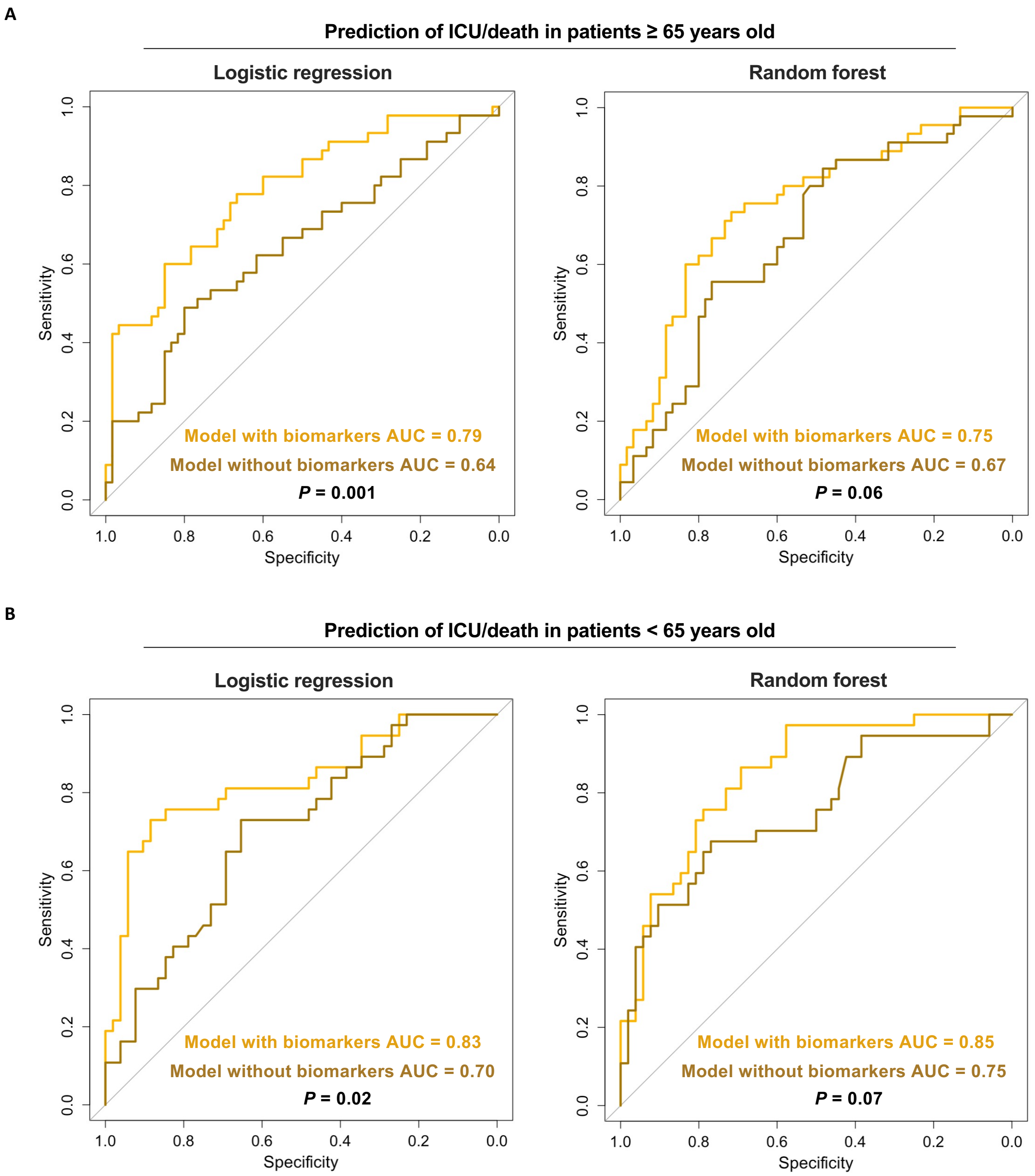

The figure shows the receiver operating characteristic curve and corresponding area under the curve (AUC) for the best logistic regression (left) and random forest (right) models for predicting ICU/death with biomarkers (gold) and without biomarkers (bronze) in the out-of-sample patients stratified by age: 65 or older (A) and younger than 65 (B).

S6 Fig. Prediction of ICU/death in out-of-sample patients stratified by gender.

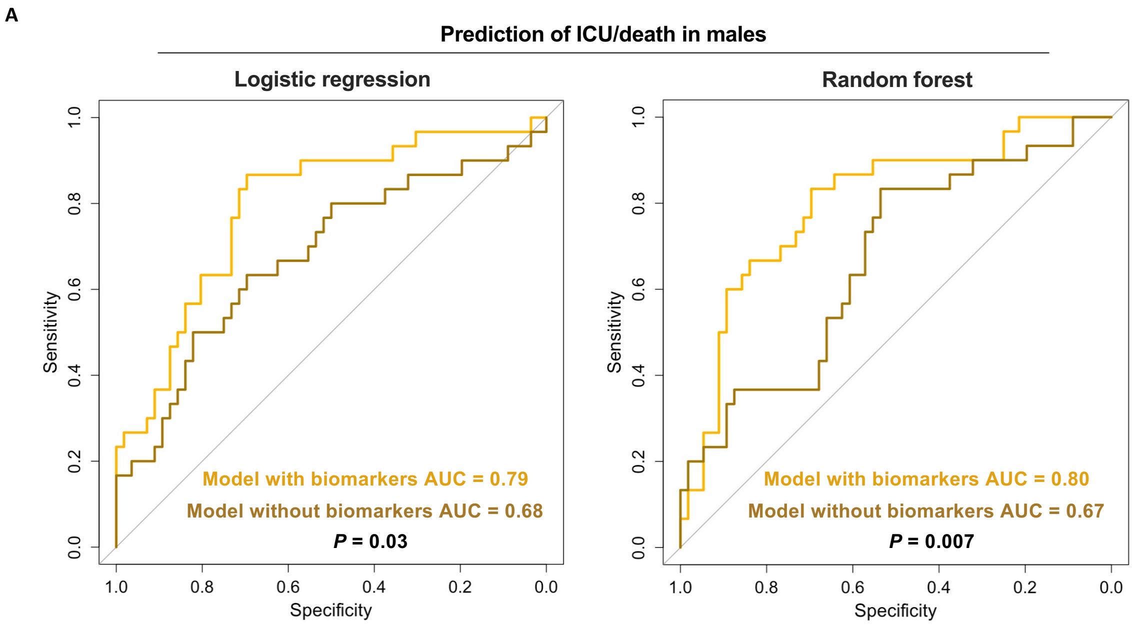

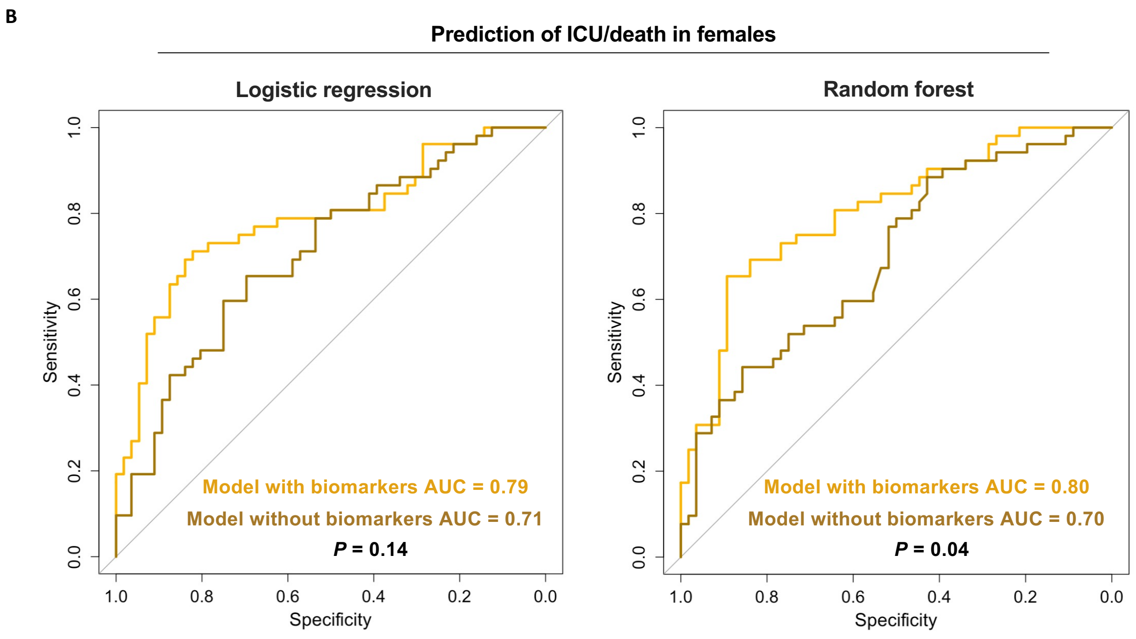

The figure shows the receiver operating characteristic curve and corresponding area under the curve (AUC) for the best logistic regression (left) and random forest (right) models for predicting ICU/death with biomarkers (gold) and without biomarkers (bronze) in the out-of-sample patients stratified by gender: male (A) and female (B).

S7 Fig. Prediction of ICU/death in out-of-sample patients in most prevalent race/ethnicity groups.

A

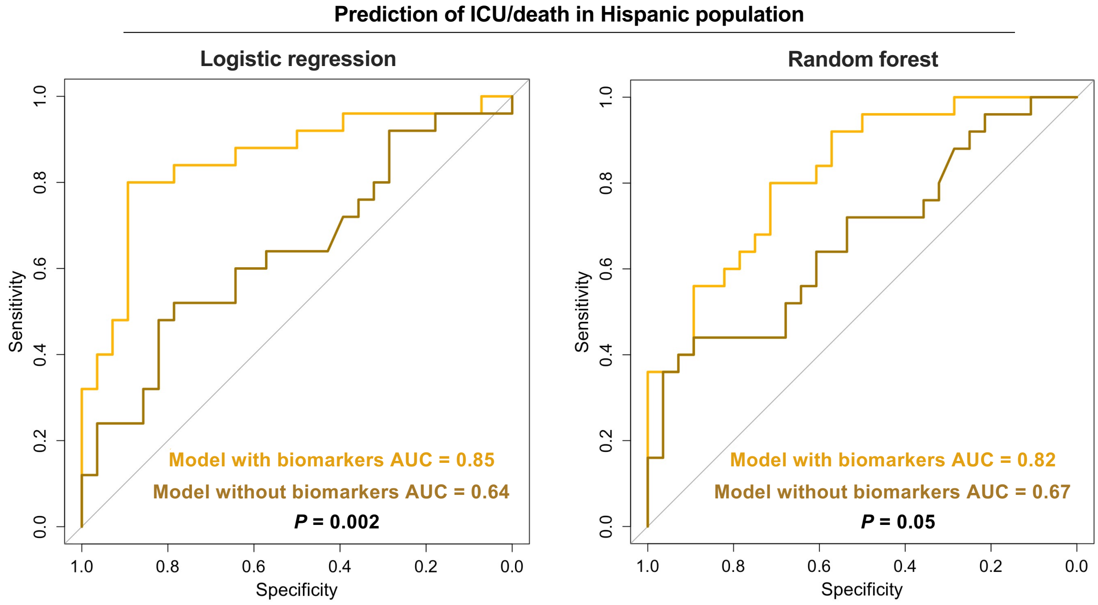

B

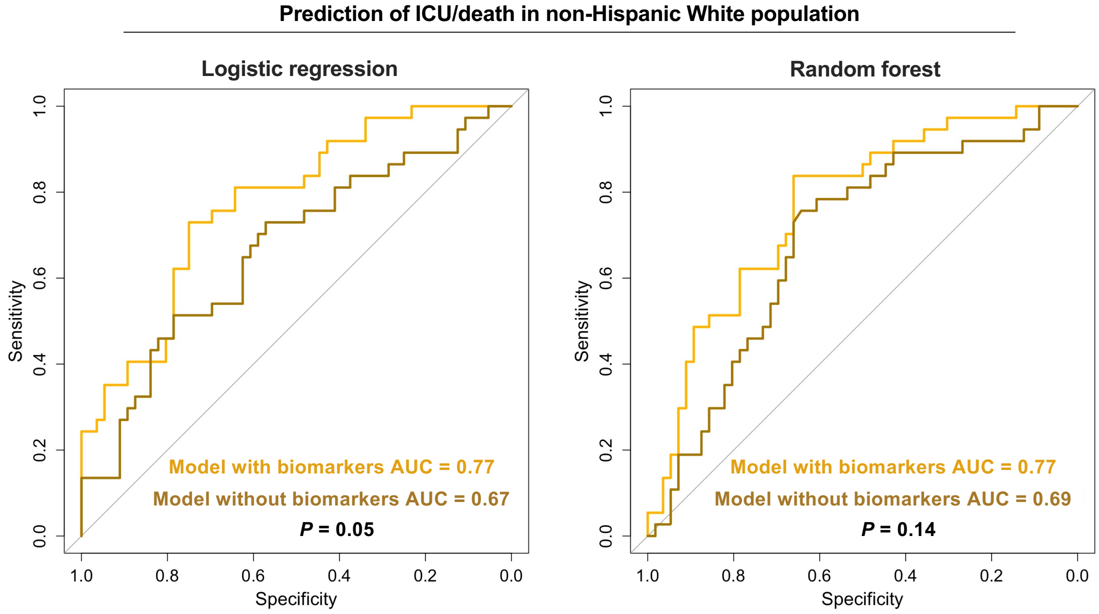

The figure shows the receiver operating characteristic curve and corresponding area under the curve (AUC) for the best logistic regression (left) and random forest (right) models for predicting ICU/death with biomarkers (gold) and without biomarkers (bronze) in the out-of-sample patients with a self-reported race/ethnicity of Hispanic (A) and non-Hispanic White (B).

S8 Fig. Prediction of ICU admission in out-of-sample patients.

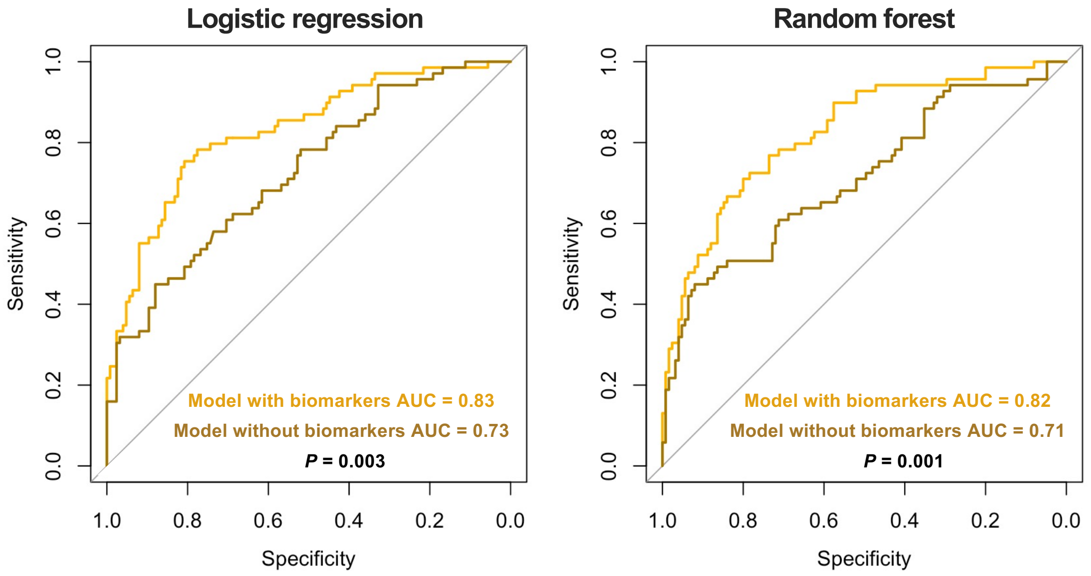

The figure shows the receiver operating characteristic curve and corresponding area under the curve (AUC) for the best logistic regression (left) and random forest (right) models for predicting ICU admission with biomarkers (gold) and without biomarkers (bronze) in the out-of-sample patients.

S9 Fig. Prediction of death in out-of-sample patients.

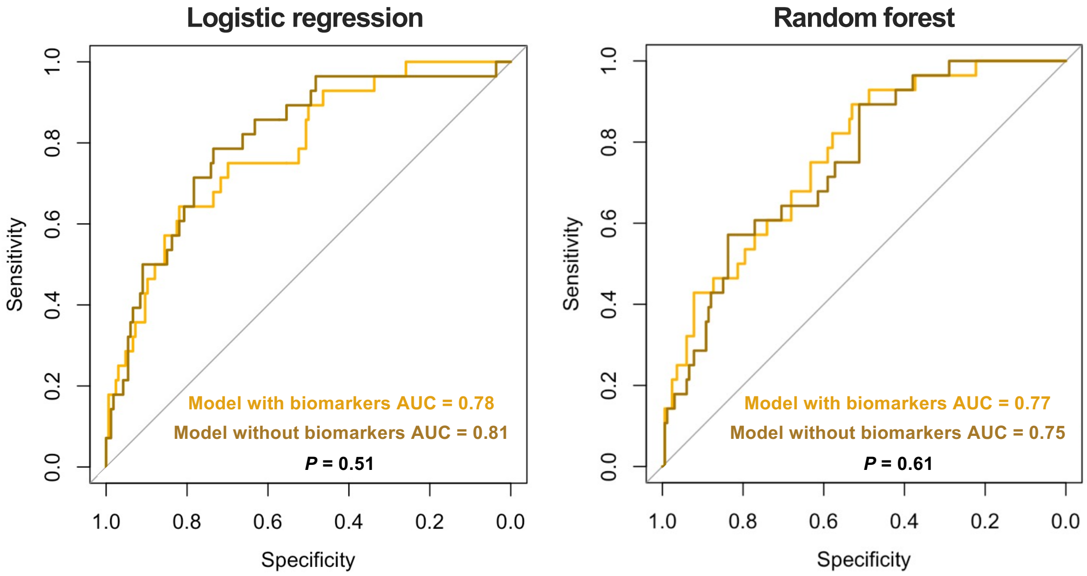

The figure shows the receiver operating characteristic curve and corresponding area under the curve (AUC) for the best logistic regression (left) and random forest (right) models for predicting death with biomarkers (gold) and without biomarkers (bronze) in the out-of-sample patients.

S1 Table. Association of cardiometabolic biomarkers and hospital labs with ICU/death.

Biomarker/hospital lab	unit	OR per SD (95% CI)	OR per unit (95% CI)	*P*
TRAIL-R2	pg/mL	5.2 (3.4 - 8.1)	4.4 (3.0 - 6.5)	1E-14
IL-4RA	pg/mL	3.3 (2.3 - 4.6)	4.0 (2.7 - 6.1)	6E-12
IL6	pg/mL	3.1 (2.2 - 4.3)	1.9 (1.6 - 2.3)	2E-11
CTSL1	pg/mL	3.1 (2.2 - 4.4)	5.6 (3.3 - 9.4)	7E-11
KIM1	pg/mL	2.9 (2.1 - 4.1)	2.2 (1.7 - 2.8)	3E-10
TNFRSF10A	pg/mL	2.7 (2.0 - 3.7)	3.5 (2.4 - 5.3)	5E-10
SCF	pg/mL	0.40 (0.29 - 0.53)	0.35 (0.25 - 0.49)	2E-09
ADM	pg/mL	2.0 (1.4 - 2.7)	1.9 (1.4 - 2.6)	2E-09
CEACAM8	pg/mL	2.5 (1.9 - 3.4)	3.0 (2.1 - 4.4)	3E-09
Procalcitonin	ng/mL	2.1 (1.3 - 3.1)	19 (2.9 - 120)	9E-09
ACE2	pg/mL	2.6 (1.9 - 3.6)	2.3 (1.8 - 3.1)	2E-08
TNFRSF11A	pg/mL	2.5 (1.8 - 3.5)	2.3 (1.7 - 3.1)	2E-08
MMP7	pg/mL	2.0 (1.5 - 2.7)	1.8 (1.4 - 2.3)	5E-08
LDH	U/L	3.2 (2.1 - 5.1)	1.0 (1.006 - 1.012)	6E-08
TM	pg/mL	2.1 (1.6 - 2.8)	3.4 (2.1 - 5.6)	2E-07
AGRP	pg/mL	2.1 (1.6 - 2.9)	2.6 (1.8 - 3.8)	4E-07
IL-1RA	pg/mL	1.5 (1.1 - 2.0)	1.8 (1.2 - 2.6)	4E-07
ADAMTS13	pg/mL	0.6 (0.45 - 0.88)	0.29 (0.12 - 0.71)	5E-07
PGF	pg/mL	2 (1.5 - 2.8)	2.4 (1.7 - 3.5)	7E-07
AST	U/L	2.2 (1.5 - 3.2)	1.0 (1.01 - 1.04)	2E-06
CCL3	pg/mL	1.9 (1.5 - 2.6)	2.3 (1.6 - 3.2)	2E-06
FABP2	pg/mL	0.52 (0.39 - 0.69)	0.66 (0.55 - 0.79)	7E-06
SORT1	pg/mL	2 (1.4 - 2.8)	3.9 (1.9 - 7.8)	7E-06
TGM2	pg/mL	0.64 (0.47 - 0.87)	0.50 (0.31 - 0.81)	7E-06
IL18	pg/mL	1.8 (1.4 - 2.4)	1.9 (1.4 - 2.4)	8E-06
DCN	pg/mL	2 (1.4 - 2.6)	3.7 (2.0 - 6.7)	1E-05
FGF-23	pg/mL	2.1 (1.5 - 2.9)	1.6 (1.3 - 1.9)	1E-05
SPON2	pg/mL	1.4 (1.1 - 1.9)	2.5 (1.1 - 5.3)	2E-05
VEGFD	pg/mL	0.47 (0.34 - 0.67)	0.31 (0.18 - 0.53)	2E-05
PAPPA	pg/mL	1.7 (1.3 - 2.2)	1.6 (1.3 - 2.1)	4E-05
PAR-1	pg/mL	1.7 (1.3 - 2.2)	3.2 (1.8 - 5.7)	6E-05
Alanine transaminase	U/L	2.1 (1.4 - 3.3)	1.0 (1.01 - 1.04)	6E-05
Dkk-1	pg/mL	1.7 (1.3 - 2.2)	2.4 (1.5 - 3.8)	8E-05
FS	pg/mL	1.7 (1.3 - 2.2)	2 (1.4 - 2.9)	0.0002
XCL1	pg/mL	1.6 (1.2 - 2.0)	1.6 (1.2 - 2.1)	0.0003
CRP	mg/L	1.8 (1.2 - 2.5)	1.0 (1.00 - 1.01)	0.0004
SOD2	pg/mL	0.57 (0.41 - 0.78)	0.038 (0.0060 - 0.24)	0.0004
REN	pg/mL	1.4 (1.1 - 1.8)	1.3 (1.1 - 1.6)	0.0005
PTX3	pg/mL	1.6 (1.2 - 2.1)	2.6 (1.5 - 4.4)	0.0005
Creatine kinase	U/L	1.8 (1.2 - 2.6)	1 (1.001 - 1.005)	0.0005
BMP-6	pg/mL	1.5 (1.2 - 1.9)	1.4 (1.2 - 1.8)	0.0008
IDUA	pg/mL	1.4 (1.1 - 1.8)	1.3 (1.1 - 1.6)	0.0008
SLAMF7	pg/mL	1.5 (1.2 - 1.9)	1.6 (1.2 - 2.0)	0.001
STK4	pg/mL	1.5 (1.2 - 1.9)	1.6 (1.2 - 2.2)	0.002
IL1RL2	pg/mL	1.5 (1.2 - 1.9)	2.0 (1.3 - 3.1)	0.002
PRSS27	pg/mL	0.68 (0.53 - 0.88)	0.55 (0.38 - 0.82)	0.002
CXCL1	pg/mL	1.5 (1.2 - 1.9)	1.6 (1.2 - 2.1)	0.002
HAOX1	pg/mL	1.5 (1.2 - 2.0)	1.2 (1.1 - 1.4)	0.002
ITGB1BP2	pg/mL	1.5 (1.2 - 1.9)	1.2 (1.1 - 1.4)	0.003
SRC	pg/mL	1.4 (1.1 - 1.8)	1.4 (1.1 - 1.8)	0.003
HO-1	pg/mL	1.5 (1.2 - 1.9)	2.0 (1.3 - 3.1)	0.003

Biomarker/hospital lab	unit	OR per SD (95% CI)	OR per unit (95% CI)	*P*
FGF-21	pg/mL	1.5 (1.2 - 2.0)	1.2 (1.1 - 1.4)	0.004
MMP12	pg/mL	1.5 (1.1 - 2.0)	1.4 (1.1 - 1.8)	0.004
PRELP	pg/mL	1.5 (1.1 - 1.9)	3.0 (1.3 - 7.2)	0.007
Glucose	mg/dL	1.3 (0.97 - 1.7)	1.0 (1.00 - 1.01)	0.007
GLO1	pg/mL	1.4 (1.1 - 1.8)	1.4 (1.1 - 1.9)	0.009
Total bilirubin	mg/dL	1.4 (1.0 - 2.0)	4.0 (1.1 - 14)	0.010
LOX-1	pg/mL	1.3 (1.1 - 1.7)	1.5 (1.1 - 2.0)	0.010
MARCO	pg/mL	1.4 (1.1 - 1.8)	2.2 (1.2 - 4.3)	0.011
Anion gap	mEq/L	1.4 (1.1 - 1.9)	1.2 (1.0 - 1.3)	0.011
TF	pg/mL	1.4 (1.1 - 1.9)	1.8 (1.2 - 2.7)	0.012
LPL	pg/mL	1.4 (1.0 - 1.8)	1.4 (1.0 - 2.0)	0.012
ESR	mm/hr	1.4 (1.0 - 1.8)	1.0 (1.00 - 1.03)	0.012
D-dimer	ng/L	1.5 (1.1 - 2.2)	1.0 (1.000 - 1.001)	0.012
Gal-9	pg/mL	1.5 (1.0 - 2.1)	2.4 (1.1 - 5.1)	0.013
THBS2	pg/mL	1.2 (0.95 - 1.6)	1.7 (0.87 - 3.3)	0.014
Ferritin	µg/L	1.5 (1.1 - 2.1)	1.0 (1.000 - 1.002)	0.016
PRSS8	pg/mL	1.4 (1.1 - 1.8)	2.2 (1.2 - 4.0)	0.019
VSIG2	pg/mL	1.3 (1.0 - 1.8)	1.4 (1.0 - 2.0)	0.020
CA5A	pg/mL	1.4 (1.1 - 1.8)	1.3 (1.0 - 1.5)	0.020
WBC	cells/nL	1.5 (1.1 - 2.1)	1.2 (1.0 - 1.4)	0.020
ALC	K/µL	0.69 (0.51 - 0.93)	0.43 (0.22 - 0.86)	0.023
CD4	pg/mL	1.3 (1.0 - 1.7)	1.6 (1.0 - 2.4)	0.025
AMBP	pg/mL	0.75 (0.58 - 0.98)	0.41 (0.18 - 0.93)	0.027
TNFRSF13B	pg/mL	1.3 (1.0 - 1.7)	1.5 (1.0 - 2.3)	0.028
NEMO	pg/mL	1.3 (1.0 - 1.7)	1.3 (1.0 - 1.5)	0.041
HB-EGF	pg/mL	1.3 (1.0 - 1.7)	1.3 (1.0 - 1.7)	0.049
Albumin	g/L	0.77 (0.58 - 1.0)	0.52 (0.26 - 1.1)	0.062
IL-27	pg/mL	1.2 (0.93 - 1.5)	1.3 (0.89 - 1.9)	0.083
CD40-L	pg/mL	1.2 (0.98 - 1.6)	1.2 (0.99 - 1.4)	0.085
Lactate	mmol/L	1.3 (0.98 - 1.7)	1.5 (0.96 - 2.4)	0.089
DECR1	pg/mL	1.2 (0.97 - 1.6)	1.1 (0.98 - 1.3)	0.11
Hemoglobin	g/dL	1.3 (0.92 - 1.7)	1.2 (0.95 - 1.4)	0.14
BUN	mg/dL	1.3 (0.95 - 1.8)	1.0 (0.99 - 1.1)	0.15
Troponin	ng/L	1.2 (0.93 - 1.6)	1.0 (1.00 - 1.03)	0.21
CCL17	pg/mL	1.2 (0.93 - 1.5)	1.1 (0.95 - 1.4)	0.24
RAGE	pg/mL	1.1 (0.88 - 1.4)	1.2 (0.82 - 1.7)	0.28
TIE2	pg/mL	1.1 (0.88 - 1.4)	1.2 (0.79 - 1.8)	0.29
GT	pg/mL	0.91 (0.71 - 1.2)	0.90 (0.69 - 1.2)	0.29
BNP	pg/mL	1.3 (0.97 - 1.7)	1.3 (0.97 - 1.7)	0.30
Platelet	U/nL	1.2 (0.90 - 1.7)	1.0 (1.00 - 1.01)	0.31
IL-17D	pg/mL	0.88 (0.67 - 1.1)	0.72 (0.37 - 1.4)	0.32
CTRC	pg/mL	1.0 (0.80 - 1.3)	1.0 (0.84 - 1.2)	0.35
SERPINA12	pg/mL	0.88 (0.69 - 1.1)	0.94 (0.83 - 1.1)	0.35
Hematocrit	%	1.1 (0.84 - 1.5)	1.0 (0.96 - 1.1)	0.39
PIgR	pg/mL	1.1 (0.86 - 1.4)	1.9 (0.37 - 9.5)	0.42
IL16	pg/mL	1.1 (0.88 - 1.4)	1.2 (0.84 - 1.7)	0.42
CD84	pg/mL	1.0 (0.82 - 1.3)	1.1 (0.69 - 1.7)	0.45
IgG-Fc-receptor-II-b	pg/mL	0.91 (0.70 - 1.2)	0.89 (0.66 - 1.2)	0.46
HSP-27	pg/mL	1.2 (0.93 - 1.6)	1.5 (0.85 - 2.6)	0.54
BOC	pg/mL	1.1 (0.85 - 1.4)	1.1 (0.76 - 1.7)	0.55
GDF-2	pg/mL	1.1 (0.83 - 1.4)	1.1 (0.84 - 1.4)	0.56

Biomarker/hospital lab	unit	OR per SD (95% CI)	OR per unit (95% CI)	*P*
MERTK	pg/mL	1.0 (0.8 - 1.3)	1.0 (0.70 - 1.5)	0.57
LEP	pg/mL	0.92 (0.67 - 1.3)	0.94 (0.75 - 1.2)	0.57
hOSCAR	pg/mL	1.1 (0.88 - 1.5)	1.6 (0.64 - 4.0)	0.58
EGFR	mL/min	0.87 (0.59 - 1.3)	0.99 (0.98 - 1.0)	0.59
PSGL-1	pg/mL	0.92 (0.7 - 1.2)	0.81 (0.40 - 1.6)	0.60
ANGPT1	pg/mL	0.95 (0.75 - 1.2)	0.93 (0.69 - 1.3)	0.66
PARP-1	pg/mL	1.0 (0.81 - 1.3)	1.0 (0.85 - 1.2)	0.68
PD-L2	pg/mL	0.93 (0.73 - 1.2)	0.87 (0.54 - 1.4)	0.73
GH	pg/mL	0.93 (0.72 - 1.2)	0.96 (0.82 - 1.1)	0.77
Creatinine	mg/dL	1.0 (0.75 - 1.4)	1.1 (0.37 - 3.3)	0.88
Alkaline phosphatase	U/L	1.1 (0.82 - 1.5)	1.0 (0.99 - 1.0)	0.88
GIF	pg/mL	1.0 (0.79 - 1.3)	1.0 (0.82 - 1.2)	0.89
THPO	pg/mL	1.0 (0.79 - 1.3)	1.0 (0.65 - 1.6)	0.95
PDGF-subunit-B	pg/mL	0.98 (0.78 - 1.2)	0.96 (0.6 - 1.5)	1.00

The table includes, for each biomarker and hospital lab, the odds ratio (OR) and *P* value resulting from a logistic regression model with ICU/death as the outcome, adjusted for the covariates: age, sex, body mass index, and self-reported ethnicity. To account for non-normality, the *P* values were calculated after applying rank-based inverse normal transformation. To preserve interpretability, the odds ratio per unit was calculated using the raw data and the odds ratio per SD was calculated from the data standardized to have a mean of 0 a standard deviation of 1. Standard deviation, SD; confidence interval, CI; lactate dehydrogenase, LDH; C-reactive protein, CRP; aspartate aminotransferase, AST; erythrocyte sedimentation rate, ERS; white blood cells, WBC; absolute lymphocyte count, ALC; blood urea nitrogen, BUN; estimated glomerular filtration rate, eGFR.

S2 Table. Ingenuity Pathway Analysis (Qiagen) of all 92 protein biomarkers.

Ingenuity canonical pathways	-log(p-value)	Ratio	Z-score	Molecules
Tumor Microenvironment Pathway	10.1	0.0585	0.905	FGF21,FGF23,IL6,LEP,LGALS9,MMP12,MMP7,PDCD1LG2,PDGFB, PGF,VEGFD
IL-10 Signaling	9.49	0.105		FCGR2B,HMOX1,IKBKG,IL18,IL1RL2,IL1RN,IL4R,IL6
Airway Pathology in Chronic Obstructive Pulmonary Disease	9.35	0.0763		AMBP,CD40LG,CXCL1,FGF21,FGF23,IL17D,IL18,IL6,LEP
Granulocyte Adhesion and Diapedesis	8.74	0.0524		CCL17,CCL3,CXCL1,IL18,IL1RL2,IL1RN,MMP12,MMP7,SELPLG,XCL1
Wound Healing Signaling Pathway	8.66	0.043	0.905	CD40LG,HBEGF,IL17D,IL18,IL1RL2,IL1RN,IL6,LEP,PDGFB,PGF,VEGFD
Osteoarthritis Pathway	7.77	0.0415	0.333	AGER,DCN,DKK1,GDF2,IL1RL2,LEP,MMP12,PGF,SERPINA12,VEGFD
Atherosclerosis Signaling	7.58	0.0606		CD40LG,F3,IL18,IL1RN,IL6,LPL,PDGFB,SELPLG
IL-17 Signaling	7.55	0.0476	1.667	CCL17,CD40LG,CXCL1,IL17D,IL18,IL6,LEP,PGF,VEGFD
Role of Macrophages, Fibroblasts and Endothelial Cells in Rheumatoid Arthritis	7.49	0.0331		DKK1,IKBKG,IL16,IL18,IL1RL2,IL1RN,IL6,PDGFB,PGF,SRC,VEGFD
Hepatic Cholestasis	7.23	0.0437		CD40LG,FABP6,IKBKG,IL17D,IL18,IL1RL2,IL1RN,IL6,LEP
HIF1α Signaling	7.09	0.0421	1.667	ADM,BMP6,HMOX1,IL6,MMP12,MMP7,PDGFB,PGF,VEGFD
IL-8 Signaling	7.07	0.0419	1.667	ANGPT1,CXCL1,HBEGF,HMOX1,IKBKG,PGF,SRC,TEK,VEGFD
Agranulocyte Adhesion and Diapedesis	7.07	0.0419		CCL17,CCL3,CXCL1,IL18,IL1RN,MMP12,MMP7,SELPLG,XCL1
Role of Osteoblasts, Osteoclasts and Chondrocytes in Rheumatoid Arthritis	6.82	0.0391		BMP6,DKK1,IKBKG,IL18,IL1RL2,IL1RN,IL6,SRC,TNFRSF11A
Th1 and Th2 Activation Pathway	6.67	0.0462		CD4,CD40LG,HAVCR1,IL18,IL27,IL4R,IL6,LGALS9
Hepatic Fibrosis Signaling Pathway	6.38	0.0256	1.508	CCL3,CD40LG,IKBKG,IL18,IL1RL2,IL1RN,LEP,PDGFB,PGF, SOD2,VEGFD
Hepatic Fibrosis / Hepatic Stellate Cell Activation	6.22	0.0404		CD40LG,IL1RL2,IL4R,IL6,LEP,PDGFB,PGF,VEGFD
Cardiac Hypertrophy Signaling (Enhanced)	6.14	0.0217	3.162	CD40LG,FGF21,FGF23,HSPB1,IKBKG,IL17D,IL18,IL1RL2,IL4R, IL6,LEP,NPPB
Bladder Cancer Signaling	5.4	0.0517		FGF21,FGF23,MMP12,MMP7,PGF,VEGFD
Acute Phase Response Signaling	5.28	0.0374	0.816	AMBP,HMOX1,IKBKG,IL18,IL1RN,IL6,SOD2
Th1 Pathway	5.25	0.0488	2.236	CD4,CD40LG,IL18,IL27,IL6,LGALS9
IL-6 Signaling	5.15	0.0469	2.449	HSPB1,IKBKG,IL18,IL1RL2,IL1RN,IL6
Clathrin-mediated Endocytosis Signaling	5.15	0.0357		F2R,FGF21,FGF23,PDGFB,PGF,SRC,VEGFD
LXR/RXR Activation	5.11	0.0462	-2.236	AMBP,IL18,IL1RL2,IL1RN,IL6,LPL
Pulmonary Healing Signaling Pathway	5.08	0.0348	1.89	CTRC,HBEGF,MMP12,MMP7,PGF,SRC,VEGFD

Ingenuity canonical pathways	-log(p-value)	Ratio	Z-score	Molecules
STAT3 Pathway	5	0.0441	1.342	BMP6,IL1RL2,IL4R,PDGFB,SRC,TNFRSF11A
Role of Pattern Recognition Receptors in Recognition of Bacteria and Viruses	4.66	0.0385		CD40LG,IL17D,IL18,IL6,LEP,PTX3
Death Receptor Signaling	4.59	0.0521	0.447	HSPB1,IKBKG,PARP1,TNFRSF10A,TNFRSF10B
HMGB1 Signaling	4.49	0.0359	2	AGER,CD40LG,IL17D,IL18,IL6,LEP
Differential Regulation of Cytokine Production in Macrophages and T Helper Cells by IL-17A and IL-17F	4.45	0.167		CCL3,CXCL1,IL6
PPAR Signaling	4.36	0.0467	-1.342	IKBKG,IL18,IL1RL2,IL1RN,PDGFB
Role of Cytokines in Mediating Communication between Immune Cells	4.36	0.0741		IL18,IL1RN,IL27,IL6
Primary Immunodeficiency Signaling	4.3	0.0714		CD4,CD40LG,IKBKG,TNFRSF13B
Erythropoietin Signaling Pathway	4.27	0.0328	-0.816	CD40LG,IL17D,IL18,IL6,LEP,SRC
Regulation Of The Epithelial Mesenchymal Transition By Growth Factors Pathway	4.16	0.0312	1	CD40LG,FGF21,FGF23,IKBKG,IL6,PDGFB
Axonal Guidance Signaling	4	0.0175		ACE2,ADAMTS13,BMP6,MMP12,MMP7,PAPPA,PDGFB,PGF,VEGFD
FXR/RXR Activation	3.85	0.0365		AMBP,FABP6,IL18,IL1RN,LPL
TREM1 Signaling	3.74	0.0513	1	CCL3,FCGR2B,IL18,IL6
Neuroinflammation Signaling Pathway	3.71	0.0212	1.134	AGER,CCL3,HMOX1,IKBKG,IL18,IL6,SOD2
Coagulation System	3.57	0.0857		F2R,F3,THBD
Role of Hypercytokinemia/hyperchemokinemia in the Pathogenesis of Influenza	3.55	0.046	2	CCL3,IL18,IL1RN,IL6
Inhibition of Matrix Metalloproteases	3.39	0.075		MMP12,MMP7,THBS2
Colorectal Cancer Metastasis Signaling	3.31	0.0217	1.633	IL6,MMP12,MMP7,PGF,SRC,VEGFD
Estrogen Receptor Signaling	3.14	0.017	0.378	LEP,MMP12,MMP7,PGF,SOD2,SRC,VEGFD
PPARα/RXRα Activation	3.1	0.025	-0.447	GH1,IKBKG,IL1RL2,IL6,LPL
Hematopoiesis from Multipotent Stem Cells	3.08	0.167		KITLG,THPO
Ephrin Receptor Signaling	3.06	0.0245		ANGPT1,PDGFB,PGF,SRC,VEGFD
Role of Tissue Factor in Cancer	3.05	0.0339		CXCL1,F3,HBEGF,SRC
p38 MAPK Signaling	3.05	0.0339	2	HSPB1,IL18,IL1RL2,IL1RN
IL-15 Production	2.99	0.0325	2	IL6,MERTK,SRC,TEK

Ingenuity canonical pathways	-log(p-value)	Ratio	Z-score	Molecules
Cholecystokinin/Gastrin-mediated Signaling	2.97	0.0323	1	GH1,IL18,IL1RN,SRC
Pancreatic Adenocarcinoma Signaling	2.92	0.0312	1	HBEGF,HMOX1,PGF,VEGFD
Dendritic Cell Maturation	2.88	0.0135	2.121	CD40LG,FCGR2B,IKBKG,IL18,IL1RL2,IL1RN,IL6,LEP
Role of PKR in Interferon Induction and Antiviral Response	2.81	0.0292		IKBKG,IL18,MARCO,PDGFB
Retinoic acid Mediated Apoptosis Signaling	2.81	0.0476		PARP1,TNFRSF10A,TNFRSF10B
Extrinsic Prothrombin Activation Pathway	2.72	0.111		F3,THBD
IL-17A Signaling in Airway Cells	2.7	0.0435		CXCL1,IKBKG,IL6
Iron homeostasis signaling pathway	2.63	0.026		BMP6,HMOX1,IL6,PDGFB
LPS/IL-1 Mediated Inhibition of RXR Function	2.61	0.0193		FABP2,FABP6,IL18,IL1RL2,IL1RN
Ovarian Cancer Signaling	2.57	0.025	1	MMP7,PGF,SRC,VEGFD
Necroptosis Signaling Pathway	2.56	0.0248	2	IKBKG,MERTK,TNFRSF10A,TNFRSF10B
Angiopoietin Signaling	2.53	0.038		ANGPT1,IKBKG,TEK
Toll-like Receptor Signaling	2.53	0.038		IKBKG,IL18,IL1RN
Aryl Hydrocarbon Receptor Signaling	2.52	0.0242		HSPB1,IL6,SRC,TGM2
Differential Regulation of Cytokine Production in Intestinal Epithelial Cells by IL-17A and IL-17F	2.51	0.087		CCL3,CXCL1
Apelin Cardiac Fibroblast Signaling Pathway	2.51	0.087		ACE2,IL6
NAD Signaling Pathway	2.48	0.0237	0	BMP6,PARP1,PDGFB,SOD2
Crosstalk between Dendritic Cells and Natural Killer Cells	2.35	0.0326		CD40LG,IL18,IL6
RANK Signaling in Osteoclasts	2.35	0.0326		IKBKG,SRC,TNFRSF11A
Glucocorticoid Receptor Signaling	2.26	0.0118		CCL3,IKBKG,IL1RL2,IL1RN,IL4R,IL6,SRC
Leukocyte Extravasation Signaling	2.23	0.0201		MMP12,MMP7,SELPLG,SRC
VEGF Signaling	2.19	0.0286		PGF,SRC,VEGFD
Pulmonary Fibrosis Idiopathic Signaling Pathway	2.18	0.0153	1.342	F2R,IL6,MMP12,MMP7,PDGFB
IL-17A Signaling in Fibroblasts	2.08	0.0526		IKBKG,IL6
Neuregulin Signaling	2.01	0.0246		DCN,HBEGF,SRC

Ingenuity canonical pathways	-log(p-value)	Ratio	Z-score	Molecules
April Mediated Signaling	2	0.0476		IKBKG,TNFRSF13B
B Cell Activating Factor Signaling	1.98	0.0465		IKBKG,TNFRSF13B
Role of IL-17F in Allergic Inflammatory Airway Diseases	1.94	0.0444		CXCL1,IL6
Coronavirus Replication Pathway	1.92	0.0435		ACE2,CTSL
Actin Cytoskeleton Signaling	1.9	0.0161		F2R,FGF21,FGF23,PDGFB
Gα12/13 Signaling	1.9	0.0224		F2R,IKBKG,SRC
Th2 Pathway	1.87	0.0219		CD4,HAVCR1,IL4R
p70S6K Signaling	1.86	0.0217		F2R,IL4R,SRC
MSP-RON Signaling In Cancer Cells Pathway	1.86	0.0217		PGF,SRC,VEGFD
FAT10 Cancer Signaling Pathway	1.85	0.04		IKBKG,IL6
IL-12 Signaling and Production in Macrophages	1.85	0.0214		CD40LG,IKBKG,IL18
Systemic Lupus Erythematosus In B Cell Signaling Pathway	1.79	0.00962	1.89	CD40LG,FCGR2B,IL17D,IL18,IL6,LEP,SRC
PI3K Signaling in B Lymphocytes	1.79	0.0203		FCGR2B,IKBKG,IL4R
Sperm Motility	1.78	0.0148		MERTK,NPPB,SRC,TEK
White Adipose Tissue Browning Pathway	1.77	0.02		FGF21,LEP,NPPB
NF-κB Signaling	1.77	0.0105	1.633	CD40LG,GH1,IKBKG,IL18,IL1RN,TNFRSF11A
Factors Promoting Cardiogenesis in Vertebrates	1.75	0.0197		BMP6,DKK1,NPPB
Endocannabinoid Cancer Inhibition Pathway	1.75	0.0197		PGF,SRC,VEGFD
Lymphotoxin β Receptor Signaling	1.74	0.0351		CXCL1,IKBKG
SPINK1 Pancreatic Cancer Pathway	1.7	0.0333		CTRC,MMP12
Oxytocin Signaling Pathway	1.69	0.0139	2	HSPB1,IL6,LEP,LPL
Activation of IRF by Cytosolic Pattern Recognition Receptors	1.64	0.0308		IKBKG,IL6
Senescence Pathway	1.62	0.0132	2	IKBKG,IL6,PARP1,SOD2
CD40 Signaling	1.61	0.0299		CD40LG,IKBKG
WNT/β-catenin Signaling	1.6	0.0172		DKK1,MMP7,SRC

Ingenuity canonical pathways	-log(p-value)	Ratio	Z-score	Molecules
Methylglyoxal Degradation I	1.55	0.125		GLO1
Macropinocytosis Signaling	1.51	0.0263		PDGFB,SRC
Phospholipases	1.5	0.026		HMOX1,LPL
Pathogenesis of Multiple Sclerosis	1.49	0.111		CCL3
Altered T Cell and B Cell Signaling in Rheumatoid Arthritis	1.49	0.0102		CD40LG,IL18,IL1RN,IL6,TNFRSF13B
NF-κB Activation by Viruses	1.48	0.0253		CD4,IKBKG
Leptin Signaling in Obesity	1.47	0.025		AGRP,LEP
Superoxide Radicals Degradation	1.45	0.1		SOD2
BEX2 Signaling Pathway	1.44	0.0241		PGF,VEGFD
PEDF Signaling	1.43	0.0238		IKBKG,SOD2
Coronavirus Pathogenesis Pathway	1.43	0.0148		ACE2,CTSL,IL6
PI3K/AKT Signaling	1.42	0.0147		IKBKG,IL1RL2,IL4R
Heme Degradation	1.41	0.0909		HMOX1
Adrenomedullin signaling pathway	1.41	0.0145		ADM,IL18,IL1RN
FGF Signaling	1.4	0.0227		FGF21,FGF23
BMP signaling pathway	1.4	0.0227		BMP6,FST
VEGF Family Ligand-Receptor Interactions	1.38	0.0222		PGF,VEGFD
PDGF Signaling	1.36	0.0217		PDGFB,SRC
mTOR Signaling	1.35	0.0138		HMOX1,PGF,VEGFD
p53 Signaling	1.31	0.0204		TNFRSF10A,TNFRSF10B
Melanocyte Development and Pigmentation Signaling	1.31	0.0204		KITLG,SRC
DNA Double-Strand Break Repair by Non-Homologous End Joining	1.31	0.0714		PARP1
Role of IL-17A in Psoriasis	1.31	0.0714		CXCL1
HER-2 Signaling in Breast Cancer	1.31	0.0132		HBEGF,IKBKG,SRC
Fcγ Receptor-mediated Phagocytosis in Macrophages and Monocytes	1.3	0.02		HMOX1,SRC

Ingenuity canonical pathways	-log(p-value)	Ratio	Z-score	Molecules
Communication between Innate and Adaptive Immune Cells	1.3	0.00753		CCL3,CD4,CD40LG,IL18,IL1RN,IL6,TNFRSF13B
Apoptosis Signaling	1.27	0.0192		IKBKG,PARP1
Granzyme B Signaling	1.25	0.0625		PARP1
Breast Cancer Regulation by Stathmin1	1.19	0.0084	0.447	BMP6,F2R,PDGFB,PGF,VEGFD
Antioxidant Action of Vitamin C	1.17	0.0169		HMOX1,IKBKG
Amyotrophic Lateral Sclerosis Signaling	1.13	0.0161		PGF,VEGFD
Systemic Lupus Erythematosus Signaling	1.12	0.00799		CD40LG,FCGR2B,IL18,IL1RN,IL6
Neuroprotective Role of THOP1 in Alzheimer's Disease	1.12	0.0159		PRSS27,PRSS8
Inflammasome pathway	1.12	0.0455		IL18
Cardiomyocyte Differentiation via BMP Receptors	1.1	0.0435		NPPB
Dermatan Sulfate Degradation (Metazoa)	1.1	0.0435		IDUA
Nitric Oxide Signaling in the Cardiovascular System	1.09	0.0152		PGF,VEGFD
Choline Biosynthesis III	1.08	0.0417		HMOX1
Role of JAK family kinases in IL-6-type Cytokine Signaling	1.06	0.04		IL6
Adipogenesis pathway	1.06	0.0147		LEP,LPL
IL-17A Signaling in Gastric Cells	1.05	0.0385		CXCL1
G Beta Gamma Signaling	1.05	0.0144		HBEGF,SRC
T Helper Cell Differentiation	1.04	0.00847		CD40LG,IL18,IL4R,IL6
Apelin Endothelial Signaling Pathway	0.996	0.0134		ANGPT1,TEK
PTEN Signaling	0.983	0.0132		IKBKG,TNFRSF11A
TNFR2 Signaling	0.963	0.0312		IKBKG
Phagosome Formation	0.959	0.00712	1.342	F2R,FCGR2B,HMOX1,MARCO,SRC
Epithelial Adherens Junction Signaling	0.947	0.0125		SRC,STK4
4-1BB Signaling in T Lymphocytes	0.936	0.0294		IKBKG
Role of JAK2 in Hormone-like Cytokine Signaling	0.936	0.0294		GH1

Ingenuity canonical pathways	-log(p-value)	Ratio	Z-score	Molecules
Ferroptosis Signaling Pathway	0.936	0.0123		HMOX1,HSPB1
HOTAIR Regulatory Pathway	0.924	0.0121		MMP12,MMP7
TWEAK Signaling	0.903	0.027		IKBKG
Human Embryonic Stem Cell Pluripotency	0.896	0.0117		BMP6,PDGFB
eNOS Signaling	0.893	0.0116		PGF,VEGFD
Glioblastoma Multiforme Signaling	0.876	0.0114		PDGFB,SRC
CXCR4 Signaling	0.873	0.0113		CD4,SRC
Gαq Signaling	0.87	0.0112		HMOX1,IKBKG
Intrinsic Prothrombin Activation Pathway	0.842	0.0233		THBD
BER (Base Excision Repair) Pathway	0.833	0.0227		PARP1
Role of RIG1-like Receptors in Antiviral Innate Immunity	0.815	0.0217		IKBKG
Role of OCT4 in Mammalian Embryonic Stem Cell Pluripotency	0.815	0.0217		PARP1
Regulation of the Epithelial-Mesenchymal Transition Pathway	0.807	0.0103		FGF21,FGF23
GNRH Signaling	0.804	0.0102		HBEGF,SRC
ILK Signaling	0.79	0.01		PGF,VEGFD
iNOS Signaling	0.783	0.02		IKBKG
MYC Mediated Apoptosis Signaling	0.775	0.0196		IKBKG
UVC-Induced MAPK Signaling	0.775	0.0196		SRC
Endothelin-1 Signaling	0.767	0.00966		HMOX1,SRC
TNFR1 Signaling	0.767	0.0192		IKBKG
RAR Activation	0.754	0.00948		PARP1,SRC
CREB Signaling in Neurons	0.747	0.00654	1	BMP6,F2R,PDGFB,TNFRSF11A
Retinol Biosynthesis	0.745	0.0182		LPL
Integrin Signaling	0.745	0.00935		PDGFB,SRC
Thrombin Signaling	0.733	0.00917		F2R,SRC

Ingenuity canonical pathways	-log(p-value)	Ratio	Z-score	Molecules
ERK/MAPK Signaling	0.733	0.00917		HSPB1,SRC
CD27 Signaling in Lymphocytes	0.73	0.0175		IKBKG
Role of IL-17A in Arthritis	0.717	0.0169		CXCL1
EGF Signaling	0.717	0.0169		SRC
Autophagy	0.714	0.00893		BMP6,PDGFB
Role of NFAT in Cardiac Hypertrophy	0.699	0.00873		IL6,SRC
Triacylglycerol Degradation	0.69	0.0159		LPL
Systemic Lupus Erythematosus In T Cell Signaling Pathway	0.69	0.00619	0	CD40LG,IL6,LEP,SELPLG
Induction of Apoptosis by HIV1	0.68	0.0154		IKBKG
NRF2-mediated Oxidative Stress Response	0.678	0.00844		HMOX1,SOD2
Hematopoiesis from Pluripotent Stem Cells	0.672	0.00679		CD4,IL6,KITLG
Thrombopoietin Signaling	0.668	0.0149		THPO
Graft-versus-Host Disease Signaling	0.662	0.00671		IL18,IL1RN,IL6
Remodeling of Epithelial Adherens Junctions	0.662	0.0147		SRC
Role of JAK1 and JAK3 in γc Cytokine Signaling	0.656	0.0145		IL4R
SPINK1 General Cancer Pathway	0.656	0.0145		IL6
PXR/RXR Activation	0.652	0.0143		IL6
Agrin Interactions at Neuromuscular Junction	0.646	0.0141		SRC
Basal Cell Carcinoma Signaling	0.635	0.0137		BMP6
ERK5 Signaling	0.635	0.0137		SRC
Phospholipase C Signaling	0.631	0.00584		FCGR2B,HMOX1,SRC,TGM2
Glioma Invasiveness Signaling	0.629	0.0135		F2R
Growth Hormone Signaling	0.625	0.0133		GH1
Caveolar-mediated Endocytosis Signaling	0.614	0.013		SRC
VDR/RXR Activation	0.606	0.0127		THBD

Ingenuity canonical pathways	-log(p-value)	Ratio	Z-score	Molecules
Role of MAPK Signaling in Inhibiting the Pathogenesis of Influenza	0.6	0.0125		IL6
Cardiac Hypertrophy Signaling	0.597	0.00743		HSPB1,IL6
Maturity Onset Diabetes of Young (MODY) Signaling	0.595	0.0123		FABP2
Antiproliferative Role of Somatostatin Receptor 2	0.595	0.0123		SRC
Renal Cell Carcinoma Signaling	0.592	0.0122		PDGFB
JAK/STAT Signaling	0.592	0.0122		IL6
Estrogen-Dependent Breast Cancer Signaling	0.587	0.012		SRC
Chemokine Signaling	0.582	0.0119		SRC
LPS-stimulated MAPK Signaling	0.577	0.0118		IKBKG
HIPPO signaling	0.573	0.0116		STK4
Xenobiotic Metabolism AHR Signaling Pathway	0.565	0.0114		IL6
FcγRIIB Signaling in B Lymphocytes	0.561	0.0112		FCGR2B
Regulation of Cellular Mechanics by Calpain Protease	0.556	0.0111		SRC
TR/RXR Activation	0.553	0.011		GH1
ICOS-ICOSL Signaling in T Helper Cells	0.551	0.00586		CD4,CD40LG,IKBKG
Xenobiotic Metabolism Signaling	0.545	0.00683		HMOX1,IL6
Insulin Secretion Signaling Pathway	0.545	0.00683		GH1,SRC
Acute Myeloid Leukemia Signaling	0.541	0.0106		KITLG
ERBB Signaling	0.541	0.0106		HBEGF
IL-4 Signaling	0.536	0.0105		IL4R
Pyroptosis Signaling Pathway	0.533	0.0104		IL18
Small Cell Lung Cancer Signaling	0.521	0.0101		IKBKG
IL-1 Signaling	0.521	0.0101		IKBKG
UVA-Induced MAPK Signaling	0.521	0.0101		PARP1
Non-Small Cell Lung Cancer Signaling	0.519	0.01		STK4

Ingenuity canonical pathways	-log(p-value)	Ratio	Z-score	Molecules
Neuropathic Pain Signaling In Dorsal Horn Neurons	0.5	0.00952		SRC
Chronic Myeloid Leukemia Signaling	0.493	0.00935		IKBKG
PD-1, PD-L1 cancer immunotherapy pathway	0.493	0.00935		PDCD1LG2
Paxillin Signaling	0.491	0.00926		SRC
Synaptogenesis Signaling Pathway	0.491	0.00621		SRC,THBS2
Sirtuin Signaling Pathway	0.485	0.00615		PARP1,SOD2
Virus Entry via Endocytic Pathways	0.484	0.00909		SRC
T Cell Exhaustion Signaling Pathway	0.471	0.00526		IL6,LGALS9,PDCD1LG2
TEC Kinase Signaling	0.456	0.00515		SRC,TNFRSF10A,TNFRSF10B
Role of NANOG in Mammalian Embryonic Stem Cell Pluripotency	0.453	0.00833		BMP6
FAK Signaling	0.453	0.00833		SRC
PAK Signaling	0.453	0.00833		PDGFB
MSP-RON Signaling In Macrophages Pathway	0.453	0.00833		IKBKG
Sphingosine-1-phosphate Signaling	0.442	0.00806		PDGFB
NGF Signaling	0.442	0.00806		IKBKG
Role of NFAT in Regulation of the Immune Response	0.44	0.00504		CD4,FCGR2B,IKBKG
Endocannabinoid Developing Neuron Pathway	0.437	0.00794		SRC
Reelin Signaling in Neurons	0.434	0.00787		SRC
Gαs Signaling	0.434	0.00787		SRC
14-3-3-mediated Signaling	0.428	0.00775		SRC
Renin-Angiotensin Signaling	0.424	0.00763		REN
Glioma Signaling	0.42	0.00758		PDGFB
HGF Signaling	0.413	0.00741		IL6
Gαi Signaling	0.394	0.00699		SRC
Xenobiotic Metabolism General Signaling Pathway	0.384	0.0068		HMOX1

Ingenuity canonical pathways	-log(p-value)	Ratio	Z-score	Molecules
Inhibition of ARE-Mediated mRNA Degradation Pathway	0.355	0.00621		CD40LG
Phagosome Maturation	0.345	0.00602		CTSL
Androgen Signaling	0.333	0.00581		SRC
Germ Cell-Sertoli Cell Junction Signaling	0.331	0.00575		SRC
Aldosterone Signaling in Epithelial Cells	0.331	0.00575		HSPB1
Type II Diabetes Mellitus Signaling	0.331	0.00575		IKBKG
G-Protein Coupled Receptor Signaling	0.323	0.00421		F2R,IKBKG,SRC
Molecular Mechanisms of Cancer	0.31	0.0044		BMP6,SRC
Mitochondrial Dysfunction	0.306	0.00532		SOD2
Xenobiotic Metabolism CAR Signaling Pathway	0.303	0.00526		SRC
Production of Nitric Oxide and Reactive Oxygen Species in Macrophages	0.292	0.00508		IKBKG
Natural Killer Cell Signaling	0.282	0.0049		IL18
Gap Junction Signaling	0.272	0.00474		SRC
Sertoli Cell-Sertoli Cell Junction Signaling	0.268	0.00467		SRC
CCR5 Signaling in Macrophages	0.264	0.00397		CCL3,CD4
RHOGDI Signaling	0.259	0.00452		SRC
Gustation Pathway	0.256	0.00448		LPL
CD28 Signaling in T Helper Cells	0.249	0.00383		CD4,IKBKG
cAMP-mediated signaling	0.239	0.00422		SRC
AMPK Signaling	0.216	0.00388		LEP
Circadian Rhythm Signaling	0	0.00358		SRC
Huntington's Disease Signaling	0	0.00348		TGM2
Calcium-induced T Lymphocyte Apoptosis	0	0.00216		CD4
Type I Diabetes Mellitus Signaling	0	0.00196		IKBKG
Allograft Rejection Signaling	0	0.00206		CD40LG

Ingenuity canonical pathways	-log(p-value)	Ratio	Z-score	Molecules
Autoimmune Thyroid Disease Signaling	0	0.00219		CD40LG
CDC42 Signaling	0	0.00173		SRC
Regulation of IL-2 Expression in Activated and Anergic T Lymphocytes	0	0.00216		IKBKG
PKCθ Signaling in T Lymphocytes	0	0.00356		CD4,IKBKG
OX40 Signaling Pathway	0	0.00209		CD4
Protein Ubiquitination Pathway	0	0.00358		HSPB1
B Cell Receptor Signaling	0	0.00315		FCGR2B,IKBKG
T Cell Receptor Signaling	0	0.00323		CD4,IKBKG
Opioid Signaling Pathway	0	0.00347		SRC
Th17 Activation Pathway	0	0.00206		IL6

S3 Table. Top networks identified in Ingenuity Pathway Analysis (Qiagen).

Molecules in network	Score	Focus molecules	Top diseases and functions
Abl1/2,ACE2,Alpha 1 antitrypsin,AMBP,BMP,BMP6,chymotrypsin, Collagen type II,Cpla2,Cr3,CTRC,DCN,Ecm,elastase,ERK1/2, farnesyl transferase,Fcer1,Fibrin,FST,Glycoprotein 1B,growth factor,LDL-cholesterol,MMP12,MMP7,NADPH oxidase,OLR1, OSCAR,PGF,PKC alpha/beta,secreted MMP,Serine Protease, SERPINA12,SMAD1/5,THBS2,VEGFD	27	14	[Cardiovascular System Development and Function, Organismal Development, Organismal Injury and Abnormalities]
ADRB,AGER,Alp,arginase,collagenase,CTSL,cytochrome C, cytochrome-c oxidase,FABP2,Fgf,FGF21,FGF23,Fgfr,GDF2, glutathione peroxidase,hexokinase,HMOX1,Hsp27,Hsp70,IL1, LDL,LEP,LPL,NFkB (complex),Ngf,Nos,PRELP,Proinsulin, PRSS27,Sod,SOD2,SORT1,STK4,Tgf beta,YAP/TAZ	27	14	[Lipid Metabolism, Protein Synthesis, Small Molecule Biochemistry]
26s Proteasome,AMPK,C1q,Complement,Fc gamma receptor, FCGR2B,FIBRINOGEN (family),HAO1,Hif1,HISTONE,Histone H2b,Hsp90,Iga,IgG,IgG1,IgG2a,Igg3,Igm,IKBKG,IL18, Immunoglobulin,ITGB1BP2,MERTK,P glycoprotein,p70 S6k, PARP,PARP1,PIGR,PRKAA,PRSS8,PTX3,SRC,TEK,TNFRSF13B, Ubiquitin	25	13	[Cell-To-Cell Signaling and Interaction, Cellular Movement, Immune Cell Trafficking]
AGRP,collagen type i (family),cytokine receptor,Fcgr2,Fcgr3, GOT,HDL,Ifn,IFN Beta,IFN type 1,Ifnar,Ikb,Ikk (family),IL-17f dimer,IL-1R,IL12 (complex),IL17a dimer,IL17D,IL1RL2,IL1RN, IL23,IL27,IL6,JAK,MARCO,Na ,K -ATPase,NFkB (family),NPPB, PDCD1LG2,Pro-inflammatory Cytokine,SELPLG,SPON2,STAT, TH17 Cytokine,Tlr	20	11	[Cell Death and Survival, Cellular Function and Maintenance, Inflammatory Response]
Adaptor protein 1,ANGPT1,Ap1,CCL17,CCL3,CD4,CD40LG, CXCL1,cytokine,Focal adhesion kinase,HBEGF,HLA-DR,Ifn gamma,Ige,IL12 (family),IL4R,Interferon alpha,LGALS9,Mapk, MHC Class I (complex),NFAT (complex),P38 MAPK,p85 (pik3r), PI3K (complex),PI3K p85,PLC gamma,RAS,RNA polymerase II, Sfk,STAT5a/b,TH2 Cytokine,TNFRSF11A,VAV,Vegf,Vla-4	18	10	[Cellular Movement, Hematological System Development and Function, Immune Cell Trafficking]

Molecules in network	Score	Focus molecules	Top diseases and functions
adhesion molecule,Akt,CD84,collagen,Collagen type IV,CPT1, creatine kinase,cyclooxygenase,DECR1,estrogen receptor, FABP6,Fibrinogen,GH1,GPIIB-IIIA,growth factor receptor,Growth hormone,H/K/NRAS,HDL-cholesterol,Histone H1,JINK1/2,KITLG, N-cor,Nr1h,PAPPA,Pdgf (complex),Pdgf Ab,PDGF BB,PDGFB, Rar,ROCK,Rxr,SAA,Shc,TGM2,THPO	15	9	[Cellular Function and Maintenance, Cellular Growth and Proliferation, Hematological System Development and Function]
ADCY,Calmodulin,chemokine,Collagen(s),DISC,DKK1,F2R,F3,G protein,G protein alphai,G-protein beta,G-protein gamma,Gpcr, Gαi/o,HAVCR1,IL16,Integrin,Jnk,JUN/JUNB/JUND, Metalloprotease,Mmp,Pde,Pdgfr,PLC,Rab5,Rac,REN,Rsk,Sapk, Smad2/3,Tnf (family),TNFRSF10A,TNFRSF10B,TSH, voltage-gated calcium channel	13	8	[Cell-To-Cell Signaling and Interaction, Cellular Movement, Immune Cell Trafficking]
ACE2,ADAMTS13,androstenediol,BOC,CA5A,CDKN1A,CEACAM5, CEACAM8,Collagen Alpha1,CSF3,D-glucose,EIF4EBP3, enterolactone,EP300,ERAP2,ESR1,FKHR,hemoglobin,IgG2b, mir-144,miR-18a-5p (and other miRNAs w/seed AAGGUGC), mir-873,MMP28,MTOR,PROK2,RTP4,SLC9B2,Stat1/3,STAT3, THBD,tretinoin,Trp53cor1,trypsin,VSIG2,VTCN1	11	7	[Cellular Development, Cellular Movement, Immune Cell Trafficking]
Actin,ADM,BCR (complex),CALC,Calcineurin protein(s),calpain, caspase,CD3,CG,Cofilin,Collagen type I (complex),Creb,Cyclin A, Dynamin,ERK,FSH,GLO1,Gsk3,Laminin (complex),Ldh (complex),Lh,MAP2K1/2,Mlc,Nfat (family),Notch,PI3K (family),Pka,Pkc(s),PTK,Raf,Rap1,Ras homolog,Rb,SLAMF7,TCR	4	3	[Cardiac Congestive Cardiac Failure, Cardiovascular Disease, Cardiovascular System Development and Function]
ALT,B4GALT6,CBLIF,CD163L1,CD80/CD86,CDKN2B-AS1,Cebp, CLEC4D,DANCR,FCGR1B,histamine,IFNG,IL17D,IL26,immune complex,Lfa-1,LILRA2,LILRA4,miR-511-5p (miRNAs w/seed UGUCUUU),mir-708,neopterin,PGLYRP2,PGLYRP4,PILRB,Ppbp, SCIMP,sesamol,SLC5A2,TAC4,TNF,TRIL,ubiquinone 9,vanillic acid,VTCN1,XCL1	4	3	[Cell-To-Cell Signaling and Interaction, Lipid Metabolism, Small Molecule Biochemistry]

Molecules in network	Score	Focus molecules	Top diseases and functions
chondroitin sulfate B,CIT,DDX58,Dermatan Sulfate Degradation (Metazoa),FBXO6,ganglioside GM3,glycosaminoglycan,heparan sulfate,IDUA,KIF23,L-iduronidase,MPP3,OBSL1,SCGB2A2,sulfate	1	1	[Developmental Disorder, Hereditary Disorder, Metabolic Disease]
Adaptor protein 2,ADGRB1,AFAP1L2,Alpha catenin,Ck2,ELP2, F Actin,Histone h3,Histone h4,HPGDS,HSPB1,IKK (complex), IL17B,Insulin,MAOA,Mek,MHC Class II (complex),MIR124, MTORC1,NCAM2,NDRG2,NKD1,P110,PDK,RSPO1,SEMA3E, SEMA6D,Sh2b3,Sos,SRC (family),TEC/BTK/ITK/TXK/BMX,TH1 Cytokine,TLR2/TLR4,TNFRSF6B,tyrosine kinase	1	1	[Cardiovascular System Development and Function, Cell Cycle, Cellular Movement]

S4 Table. Logistic regression coefficients of variables included in best models for predicting ICU/death with and without biomarkers.

Logistic regression									
**Best model without biomarkers**									
	**In-sample**			**Out-of-sample**			**Total**		
	**β**	**β 95% CI**	***P***	**β**	**β 95% CI**	***P***	**β**	**β 95% CI**	***P***
Procalcitonin	0.49	(-0.01, 2)	0.33	-0.042	(-0.2, 0.09)	0.60	0.045	(-0.01, 0.1)	0.16
LDH	0.0053	(3E-03, 8E-03)	3E-05	0.006	(3E-03, 9E-03)	1E-04	0.0054	(4E-03, 7E-03)	7E-09
CRP	0.0048	(5E-04, 9E-03)	0.03	0.0021	(-3E-03, 8E-03)	0.47	0.0043	(1E-03, 7E-03)	0.01
BMI	0.45	(0.2, 0.7)	0.002	0.21	(-0.1, 0.5)	0.19	0.38	(0.2, 0.6)	2E-04
WBC	0.11	(4E-03, 0.2)	0.07	0.033	(-0.06, 0.1)	0.50	0.058	(-1E-03, 0.1)	0.07
Glucose	0.0024	(-1E-03, 6E-03)	0.22	0.0018	(-1E-03, 6E-03)	0.28	0.0018	(-5E-04, 4E-03)	0.14
D-dimer	0.00031	(-5E-05, 7E-04)	0.12	-0.00016	(-4E-04, 9E-05)	0.21	-0.0000078	(-2E-04, 2E-04)	0.93
Troponin	0.00020	(-4E-03, 4E-03)	0.92	0.00012	(-4E-03, 3E-03)	0.95	0.0011	(-1E-03, 4E-03)	0.35
Hispanic	0.50	(-0.05, 1)	0.08	-0.22	(-1, 0.5)	0.57	0.24	(-0.2, 0.7)	0.26
Total bilirubin	-0.17	(-0.6, 0.1)	0.27	0.17	(-0.7, 1)	0.68	-0.067	(-0.4, 0.2)	0.60
ALC	-0.12	(-0.3, 0.2)	0.19	-0.56	(-1, 5E-03)	0.06	-0.077	(-0.2, 0.1)	0.21
**Best model with biomarkers**									
	**In-sample**			**Out-of-sample**			**Total**		
	**β**	**β 95% CI**	***P***	**β**	**β 95% CI**	***P***	**β**	**β 95% CI**	***P***
Procalcitonin	0.30	(0.01, 1)	0.38	-0.079	(-0.3, 0.1)	0.55	0.047	(-0.01, 0.1)	0.17
LDH	0.0043	(2E-03, 7E-03)	0.002	0.0041	(1E-03, 8E-03)	0.01	0.0040	(2E-03, 6E-03)	7E-05
IL-1RA	0.48	(-0.04, 1)	0.07	0.54	(-0.2, 1)	0.16	0.51	(0.1, 0.9)	0.01
CTSL1	0.43	(-0.2, 1)	0.15	0.64	(0.03, 1)	0.04	0.63	(0.2, 1)	0.003
ADAMTS13	-1.3	(-2, -0.3)	0.01	-1.5	(-3, -0.1)	0.04	-1.3	(-2, -0.5)	9E-04
VEGFD	-1.1	(-2, -0.5)	3E-04	-0.58	(-1, 0.1)	0.10	-1.0	(-1, -0.6)	5E-06
KIM1	0.38	(0.1, 0.7)	0.01	0.21	(-0.08, 0.5)	0.15	0.29	(0.1, 0.5)	0.002
ACE2	0.36	(0.03, 0.7)	0.03	0.16	(-0.2, 0.6)	0.43	0.32	(0.08, 0.6)	0.01
IL6	0.28	(0.06, 0.5)	0.02	0.10	(-0.1, 0.3)	0.33	0.17	(0.02, 0.3)	0.03

The table shows the coefficients of each protein biomarker and hospital laboratory test included in the best logistic regression models built with and without the biomarkers. The coefficients and *P* values were estimated using the raw data for the in-sample, out-of-sample, and total patient population. Confidence interval, CI; lactate dehydrogenase, LDH; C-reactive protein, CRP; body mass index, BMI; white blood cells, WBC; absolute lymphocyte count, ALC.

S5 Table. Logistic regression coefficients of variables included in best models for predicting ICU/death with and without biomarkers in patients with blood sample collection within 14 days of presentation to care.

Logistic regression									
**Best model without biomarkers**									
	**In-sample**			**Out-of-sample**			**Total**		
	**β**	**β 95% CI**	***P***	**β**	**β 95% CI**	***P***	**β**	**β 95% CI**	***P***
Procalcitonin	0.42	(2E-03, 2)	0.41	-0.031	(-0.2, 0.1)	0.68	0.036	(-0.02, 0.1)	0.26
LDH	0.0056	(3E-03, 8E-03)	5E-06	0.0057	(3E-03, 9E-03)	2E-04	0.0054	(4E-03, 7E-03)	2E-08
CRP	0.005	(3E-04, 0.01)	0.04	0.00089	(-5E-03, 7E-03)	0.76	0.0045	(1E-03, 8E-03)	0.01
BMI	0.058	(0.01, 0.1)	0.02	0.031	(-0.02, 0.09)	0.25	0.050	(0.02, 0.09)	0.004
WBC	0.12	(1E-03, 0.3)	0.08	0.048	(-0.05, 0.2)	0.36	0.054	(-9E-03, 0.1)	0.11
Glucose	0.0026	(-2E-03, 7E-03)	0.23	0.0015	(-2E-03, 5E-03)	0.35	0.0018	(-6E-04, 4E-03)	0.15
D-dimer	0.00027	(-1E-04, 7E-04)	0.21	-0.00015	(-4E-04, 1E-04)	0.26	-0.000031	(-2E-04, 2E-04)	0.74
Troponin	-0.00039	(-5E-03, 4E-03)	0.86	-0.00026	(-5E-03, 3E-03)	0.89	0.00042	(-2E-03, 3E-03)	0.74
Hispanic	0.81	(-0.2, 2)	0.11	-0.093	(-1, 1)	0.89	0.38	(-0.4, 1)	0.32
Total bilirubin	-0.11	(-0.5, 0.2)	0.47	0.052	(-0.8, 0.9)	0.9	-0.029	(-0.3, 0.2)	0.82
ALC	-0.14	(-0.3, 0.2)	0.2	-0.54	(-1, 0.03)	0.07	-0.08	(-0.2, 0.1)	0.18
**Best model with biomarkers**									
	**In-sample**			**Out-of-sample**			**Total**		
	**β**	**β 95% CI**	***P***	**β**	**β 95% CI**	***P***	**β**	**β 95% CI**	***P***
Procalcitonin	0.48	(0.02, 1)	0.21	-0.051	(-0.3, 0.1)	0.67	0.041	(-0.02, 0.1)	0.25
LDH	0.0043	(2E-03, 7E-03)	0.004	0.0035	(3E-04, 7E-03)	0.05	0.0039	(2E-03, 6E-03)	3E-04
IL-1RA	0.20	(-0.4, 0.8)	0.52	0.59	(-0.2, 1)	0.16	0.45	(0.03, 0.9)	0.04
CTSL1	0.49	(-0.2, 1)	0.15	0.84	(0.2, 2)	0.02	0.73	(0.3, 1)	0.001
ADAMTS13	-0.87	(-2, 0.3)	0.14	-2.0	(-4, -0.5)	0.02	-1.2	(-2, -0.4)	0.004
VEGFD	-1.0	(-2, -0.4)	0.003	-0.71	(-2, 0.08)	0.09	-1.0	(-2, -0.6)	3E-05
KIM1	0.24	(-0.08, 0.6)	0.14	0.29	(-0.05, 0.6)	0.09	0.26	(0.04, 0.5)	0.02
ACE2	0.35	(-0.05, 0.8)	0.09	0.11	(-0.4, 0.6)	0.65	0.21	(-0.07, 0.5)	0.14
IL6	0.43	(0.2, 0.7)	0.002	0.059	(-0.2, 0.3)	0.59	0.20	(0.04, 0.4)	0.02

Random forest									
**Best model without biomarkers**									
	**In-sample**			**Out-of-sample**			**Total**		
	**β**	**β 95% CI**	***P***	**β**	**β 95% CI**	***P***	**β**	**β 95% CI**	***P***
CRP	0.0062	(2E-03, 0.01)	0.01	0.0025	(-3E-03, 8E-03)	0.4	0.0050	(2E-03, 8E-03)	0.003
LDH	0.0048	(2E-03, 8E-03)	0.004	0.0051	(1E-03, 9E-03)	0.009	0.0048	(3E-03, 7E-03)	3E-05
Procalcitonin	0.39	(5E-03, 1)	0.39	-0.0075	(-0.2, 0.1)	0.92	0.044	(-0.02, 0.1)	0.18
WBC	0.066	(-0.03, 0.2)	0.25	-0.0069	(-0.1, 0.09)	0.89	0.026	(-0.03, 0.09)	0.41
Lactate	0.28	(-0.2, 0.8)	0.23	0.30	(-0.09, 0.7)	0.15	0.25	(-0.01, 0.5)	0.08
D-dimer	0.00019	(-1E-04, 6E-04)	0.32	-0.00012	(-4E-04, 1E-04)	0.35	-0.000028	(-2E-04, 2E-04)	0.76
BMI	0.069	(0.02, 0.1)	0.008	0.036	(-0.02, 0.09)	0.2	0.054	(0.02, 0.09)	0.003
AST	-0.0063	(-0.02, 0.01)	0.44	0.0065	(-8E-03, 0.03)	0.45	-0.00066	(-0.01, 0.01)	0.91
Glucose	0.0023	(-2E-03, 7E-03)	0.28	0.0011	(-3E-03, 5E-03)	0.54	0.0014	(-1E-03, 4E-03)	0.29
Ferritin	0.00014	(-3E-04, 7E-04)	0.56	-0.000019	(-3E-04, 3E-04)	0.89	0.0000090	(-2E-04, 3E-04)	0.94
ALT	0.013	(-4E-03, 0.03)	0.15	-0.0096	(-0.03, 8E-03)	0.29	0.0040	(-7E-03, 0.02)	0.47
**Best model with biomarkers**									
	**In-sample**			**Out-of-sample**			**Total**		
	**β**	**β 95% CI**	***P***	**β**	**β 95% CI**	***P***	**β**	**β 95% CI**	***P***
Procalcitonin	0.48	(0.02, 1)	0.21	-0.051	(-0.3, 0.1)	0.67	0.041	(-0.02, 0.1)	0.25
LDH	0.0043	2E-03, 7E-03)	0.004	0.0035	(3E-04, 7E-03)	0.05	0.0039	(2E-03, 6E-03)	3E-04
IL-1RA	0.20	(-0.4, 0.8)	0.52	0.59	(-0.2, 1)	0.16	0.45	(0.03, 0.9)	0.04
CTSL1	0.49	(-0.2, 1)	0.15	0.84	(0.2, 2)	0.02	0.73	(0.3, 1)	0.001
ADAMTS13	-0.87	(-2, 0.3)	0.14	-2.0	(-4, -0.5)	0.02	-1.2	(-2, -0.4)	0.004
VEGFD	-1.0	(-2, -0.4)	0.003	-0.71	(-2, 0.08)	0.09	-1.0	(-2, -0.6)	3E-05
KIM1	0.24	(-0.08, 0.6)	0.14	0.29	(-0.05, 0.6)	0.09	0.26	(0.04, 0.5)	0.02
ACE2	0.35	(-0.05, 0.8)	0.09	0.11	(-0.4, 0.6)	0.65	0.21	(-0.07, 0.5)	0.14
IL6	0.43	(0.2, 0.7)	0.002	0.059	(-0.2, 0.3)	0.59	0.20	(0.04, 0.4)	0.02

The coefficients and *P* values were estimated using the raw hospital lab and biomarker measurements for the in-sample, out-of-sample, and total patient population. Confidence interval, CI; lactate dehydrogenase, LDH; C-reactive protein, CRP; body mass index, BMI; white blood cells, WBC; absolute lymphocyte count, ALC; aspartate aminotransferase, AST; alanine transaminase, ALT.

S6 Table. Performance of logistic regression models in out-of-sample patients when stratifying and adjusting by CURB-65 score.

	CURB-65 ≤ 1 (n = 104)	CURB-65 > 1 (n = 90)	Unadjusted by CURB-65	Adjusted by CURB-65
Model with biomarkers AUC	0.83	0.81	0.82	0.81
Model without biomarkers AUC	0.71	0.68	0.70	0.68
P of AUC difference	0.005	0.008	0.001	0.003

S7 Table. Logistic regression coefficients of variables included in best models for predicting ICU admission with and without biomarkers.

Logistic regression									
**Best model without biomarkers**									
	**In-sample**			**Out-of-sample**			**Total**		
	**β**	**β 95% CI**	***P***	**β**	**β 95% CI**	***P***	**β**	**β 95% CI**	***P***
Procalcitonin	0.33	(-0.01, 1)	0.28	0.0076	(-0.2, 0.1)	0.93	0.050	(-8E-03, 0.1)	0.16
LDH	0.0038	(1E-03, 7E-03)	0.004	0.0050	(2E-03, 9E-03)	0.007	0.0044	(2E-03, 6E-03)	2E-05
CRP	0.0075	(4E-03, 0.01)	3E-04	0.0023	(-3E-03, 8E-03)	0.41	6.0	(3E-03, 9E-03)	6E-05
BMI	0.069	(0.03, 0.1)	0.002	0.037	(-0.02, 0.09)	0.18	0.059	(0.03, 0.09)	4E-04
ALT	0.0086	(-5E-03, 0.03)	0.26	-0.0075	(-0.03, 0.01)	0.41	0.0048	(-5E-03, 0.01)	0.33
AST	0.0023	(-0.01, 0.02)	0.77	0.0042	(-9E-03, 0.02)	0.55	-0.00090	(-0.01, 9E-03)	0.85
Hispanic	0.85	(-9E-03, 2)	0.05	-0.18	(-2, 1)	0.79	0.45	(-0.2, 1)	0.2
Creatine kinase	-0.000054	(-5E-04, 3E-04)	0.79	-0.0011	(-3E-03, 8E-05)	0.11	-0.000088	(-5E-04, 3E-04)	0.63
Ferritin	0.00017	(-3E-04, 7E-04)	0.48	0.000070	(-2E-04, 3E-04)	0.6	0.000010	(-2E-04, 2E-04)	0.92
Glucose	0.0018	(-2E-03, 6E-03)	0.31	0.00020	(-4E-03, 3E-03)	0.91	0.00059	(-2E-03, 3E-03)	0.62
**Best model with biomarkers**									
	**In-sample**			**Out-of-sample**			**Total**		
	**β**	**β 95% CI**	***P***	**β**	**β 95% CI**	***P***	**β**	**β 95% CI**	***P***
Procalcitonin	0.16	(-0.02, 0.8)	0.54	0.0070	(-0.2, 0.2)	0.95	0.0047	(-0.01, 0.1)	0.16
LDH	0.0033	(1E-03, 6E-03)	0.009	0.0026	(-4E-04, 6E-03)	0.1	0.0029	(1E-03, 5E-03)	0.002
IL-1RA	0.21	(-0.3, 0.8)	0.45	0.64	(-0.2, 2)	0.14	0.39	(-0.01, 0.8)	0.06
CTSL1	0.76	(0.2, 1)	0.02	0.55	(-0.1, 1)	0.11	0.76	(0.3, 1)	5E-04
ADAMTS13	-1.3	(-2, -0.2)	0.02	-2.4	(-4, -0.8)	0.01	-1.5	(-2, -0.7)	4E-04
VEGFD	-1.1	(-2, -0.5)	5E-04	-0.69	(-2, 0.1)	0.11	-1.1	(-2, -0.6)	8E-06
KIM1	0.49	(0.2, 0.8)	0.001	0.28	(-0.08, 0.6)	0.12	0.37	(0.2, 0.6)	6E-04
ACE2	0.34	(-0.02, 0.7)	0.07	0.26	(-0.2, 0.7)	0.26	0.31	(0.05, 0.6)	0.02
IL6	0.27	(0.04, 0.5)	0.03	0.10	(-0.1, 0.3)	0.36	0.17	(0.01, 0.3)	0.03

Random forest									
**Best model without biomarkers**									
	**In-sample**			**Out-of-sample**			**Total**		
	**β**	**β 95% CI**	***P***	**β**	**β 95% CI**	***P***	**β**	**β 95% CI**	***P***
CRP	0.0069	(3E-03, 0.01)	0.002	0.0029	(-3E-03, 9E-03)	0.35	0.0054	(2E-03, 9E-03)	6E-04
LDH	0.0037	(1E-03, 6E-03)	0.007	0.0051	(2E-03, 9E-03)	0.006	0.0042	(2E-03, 6E-03)	4E-05
Procalcitonin	0.26	(2E-03, 0.9)	0.39	0.025	(-0.2, 0.2)	0.75	0.052	(-6E-03, 0.1)	0.14
AST	0.0030	(-0.01, 0.02)	0.7	0.0027	(-0.01, 0.02)	0.71	-0.00036	(-0.01, 0.01)	0.94
ALT	0.0072	(-7E-03, 0.02)	0.37	-0.0069	(-0.03, 0.01)	0.44	0.0034	(-6E-03, 0.01)	0.5
Ferritin	0.00016	(-3E-04, 7E-04)	0.48	0.000052	(-2E-04, 3E-04)	0.7	0.0000053	(-2E-04, 2E-04)	0.96
COPD	-0.53	(-1, 0.3)	0.2	0.40	(-0.7, 1)	0.48	-0.21	(-0.9, 0.5)	0.54
Age	0.0057	(-0.01, 0.02)	0.56	-0.014	(-0.04, 0.01)	0.26	-0.0010	(-0.01, 0.01)	0.88
Creatine kinase	-0.000097	(-6E-04, 3E-04)	0.63	-0.0014	(-3E-03, -8E-05)	0.07	-0.00012	(-5E-04, 2E-04)	0.52
Lactate	0.057	(-0.3, 0.4)	0.75	0.41	(0.03, 0.8)	0.05	0.17	(-0.05, 0.4)	0.15
WBC	0.041	(-0.03, 0.1)	0.32	-0.014	(-0.1, 0.09)	0.78	0.021	(-0.03, 0.08)	0.44
BMI	0.069	(0.03, 0.1)	0.002	0.047	(-8E-03, 0.1)	0.1	0.062	(0.03, 0.1)	2E-04
**Best model with biomarkers**									
	**In-sample**			**Out-of-sample**			**Total**		
	**β**	**β 95% CI**	***P***	**β**	**β 95% CI**	***P***	**β**	**β 95% CI**	***P***
Procalcitonin	0.16	(-0.02, 0.8)	0.54	0.0070	(-0.2, 0.2)	0.95	0.0047	(-0.01, 0.1)	0.16
LDH	0.0033	(1E-03, 6E-03)	0.009	0.0026	(-4E-04, 6E-03)	0.1	0.0029	(1E-03, 5E-03)	0.002
IL-1RA	0.21	(-0.3, 0.8)	0.45	0.64	(-0.2, 2)	0.14	0.39	(-0.01, 0.8)	0.06
CTSL1	0.76	(0.2, 1)	0.02	0.55	(-0.1, 1)	0.11	0.76	(0.3, 1)	5E-04
ADAMTS13	-1.3	(-2, -0.2)	0.02	-2.4	(-4, -0.8)	0.01	-1.5	(-2, -0.7)	4E-04
VEGFD	-1.1	(-2, -0.5)	5E-04	-0.69	(-2, 0.1)	0.11	-1.1	(-2, -0.6)	8E-06
KIM1	0.49	(0.2, 0.8)	0.001	0.28	(-0.08, 0.6)	0.12	0.37	(0.2, 0.6)	6E-04
ACE2	0.34	(-0.02, 0.7)	0.07	0.26	(-0.2, 0.7)	0.26	0.31	(0.05, 0.6)	0.02
IL6	0.27	(0.04, 0.5)	0.03	0.10	(-0.1, 0.3)	0.36	0.17	(0.01, 0.3)	0.03

The coefficients and *P* values were estimated using the raw hospital lab and biomarker measurements for the in-sample, out-of-sample, and total patient population. Confidence interval, CI; lactate dehydrogenase, LDH; C-reactive protein, CRP; body mass index, BMI; alanine transaminase, ALT; aspartate aminotransferase, AST; white blood cells, WBC; chronic obstructive pulmonary disease, COPD.

S8 Table. Logistic regression coefficients of variables included in best models for predicting death with and without biomarkers.

Logistic regression									
**Best model without biomarkers**									
	**In-sample**			**Out-of-sample**			**Total**		
	**β**	**β 95% CI**	***P***	**β**	**β 95% CI**	***P***	**β**	**β 95% CI**	***P***
Age	0.088	(0.05, 0.1)	3E-05	0.13	(0.06, 0.2)	6E-04	0.079	(0.05, 0.1)	4E-07
HFrEF	1.5	(-0.8, 4)	0.18	1.8	(-0.05, 4)	0.06	0.90	(-0.3, 2)	0.13
Non-Hispanic White	0.90	(-0.4, 2)	0.2	0.13	(-3, 3)	0.93	0.57	(-0.5, 2)	0.31
ALC	-0.047	(-0.3, 0.1)	0.58	-1.1	(-2, 0.08)	0.09	-0.082	(-0.4, 0.08)	0.53
Lactate	0.36	(-2E-03, 0.8)	0.06	-0.62	(-1, 0.08)	0.11	0.054	(-0.2, 0.3)	0.68
Procalcitonin	0.14	(0.04, 0.4)	0.13	0.14	(-0.1, 0.3)	0.15	0.11	(0.03, 0.2)	0.03
ALT	-0.0093	(-0.04, 0.01)	0.42	0.0065	(-0.03, 0.04)	0.71	-0.0045	(-0.02, 0.01)	0.58
BUN	0.028	(-0.01, 0.07)	0.17	-0.015	(-0.07, 0.04)	0.57	0.010	(-0.01, 0.03)	0.38
EGFR	0.0033	(-0.03, 0.03)	0.83	0.0097	(-0.03, 0.05)	0.66	0.0043	(-0.02, 0.03)	0.69
WBC	0.040	(-0.09, 0.1)	0.48	0.058	(-0.1, 0.2)	0.43	0.033	(-0.04, 0.1)	0.37
Creatinine	-0.38	(-1, 0.4)	0.41	-0.21	(-1, 0.5)	0.64	-0.056	(-0.6, 0.3)	0.8
Troponin	-0.0015	(-0.01, 5E-03)	0.68	0.00039	(-7E-03, 6E-03)	0.9	-0.0012	(-6E-03, 3E-03)	0.58
AST	-0.0022	(-0.02, 0.02)	0.82	-0.0045	(-0.03, 0.02)	0.69	-0.00044	(-0.01, 0.01)	0.94
Ferritin	0.00040	(-7E-06, 8E-04)	0.06	0.000037	(-2E-04, 3E-04)	0.79	0.00011	(-9E-05, 3E-04)	0.28
Albumin	-0.12	(-1, 0.8)	0.8	0.093	(-1, 1)	0.86	-0.35	(-1, 0.3)	0.26
Hematrocrit	-0.12	(-0.4, 0.2)	0.44	0.95	(0.5, 1)	7E-05	0.22	(0.02, 0.4)	0.03
LDH	0.0020	(-8E-04, 5E-03)	0.16	0.0059	(2E-03, 0.01)	0.01	0.0025	(6E-04, 4E-03)	0.01
Hemoglobin	0.23	(-0.6, 1)	0.59	-2.5	(-4, -1)	3E-04	-0.55	(-1, 0.04)	0.06
**Best model with biomarkers**									
	**In-sample**			**Out-of-sample**			**Total**		
	**β**	**β 95% CI**	***P***	**β**	**β 95% CI**	***P***	**β**	**β 95% CI**	***P***
Age	0.092	(0.06, 0.1)	2E-07	0.078	(0.03, 0.1)	0.002	0.086	(0.06, 0.1)	9E-10
HFrEF	2.5	(0.5, 4)	0.01	1.1	(-0.4, 3)	0.13	1.6	(0.5, 3)	0.004
Non-Hispanic White	0.70	(-0.5, 2)	0.26	-0.31	(-2, 2)	0.77	0.43	(-0.5, 2)	0.41
ALC	-0.038	(-0.3, 0.06)	0.6	-0.19	(-1, 0.7)	0.7	-0.042	(-0.3, 0.06)	0.59
IL-1RA	1.2	(0.2, 2)	0.03	0.22	(-0.9, 1)	0.71	0.73	(0.05, 2)	0.05
CTSL1	0.43	(-0.3, 1)	0.26	0.57	(-0.3, 1)	0.19	0.46	(-0.06, 1)	0.09
ADAMTS13	-1.0	(-2, 0.4)	0.14	0.42	(-1, 2)	0.64	-0.23	(-1, 0.8)	0.65
VEGFD	-0.46	(-1, 0.2)	0.15	-0.51	(-2, 0.5)	0.31	-0.47	(-0.9, 6E-03)	0.05
KIM1	-0.25	(-0.6, 0.09)	0.16	0.19	(-0.3, 0.6)	0.42	-0.090	(-0.4, 0.2)	0.51
ACE2	0.36	(-0.09, 0.8)	0.12	-0.17	(-0.9, 0.5)	0.63	0.17	(-0.2, 0.5)	0.34
IL6	0.28	(0.02, 0.5)	0.03	0.24	(-0.03, 0.5)	0.08	0.24	(0.07, 0.4)	0.01

Random forest									
**Best model without biomarkers**									
	**In-sample**			**Out-of-sample**			**Total**		
	**β**	**β 95% CI**	***P***	**β**	**β 95% CI**	***P***	**β**	**β 95% CI**	***P***
Age	0.089	(0.05, 0.1)	4E-05	0.14	(0.06, 0.2)	9E-04	0.077	(0.05, 0.1)	1E-06
BMI	0.056	(-1E-03, 0.1)	0.05	0.026	(-0.06, 0.1)	0.56	0.042	(-2E-03, 0.09)	0.06
HFrEF	1.6	(-0.7, 4)	0.15	2.0	(-0.05, 4)	0.06	0.95	(-0.3, 2)	0.12
Non-Hispanic White	0.87	(-0.4, 2)	0.23	0.25	(-3, 4)	0.88	0.63	(-0.4, 2)	0.26
CKD	1.0	(-6E-03, 2)	0.05	0.029	(-2, 2)	0.97	0.64	(-0.1, 1)	0.1
ALC	-0.062	(-0.3, 0.1)	0.47	-1.3	(-3, -0.06)	0.06	-0.078	(-0.4, 0.07)	0.48
Lactate	0.37	(-8E-03, 0.8)	0.06	-0.51	(-1, 0.2)	0.19	0.034	(-0.2, 0.3)	0.8
Procalcitonin	0.14	(0.04, 0.4)	0.09	0.14	(-0.2, 0.3)	0.29	0.11	(0.02, 0.2)	0.04
ALT	-0.0076	(-0.03, 0.01)	0.5	0.0022	(-0.04, 0.04)	0.91	-0.0060	(-0.02, 9E-03)	0.48
BUN	0.039	(-1E-03, 0.08)	0.07	-0.017	(-0.07, 0.04)	0.55	0.012	(-0.01, 0.04)	0.33
EGFR	0.015	(-0.02, 0.05)	0.37	0.021	(-0.03, 0.07)	0.39	0.011	(-0.01, 0.03)	0.34
WBC	0.069	(-0.06, 0.2)	0.23	0.038	(-0.1, 0.2)	0.61	0.028	(-0.05, 0.1)	0.47
Creatinine	-0.43	(-1, 0.4)	0.35	-0.29	(-2, 0.5)	0.59	-0.063	(-0.6, 0.3)	0.78
Troponin	-0.0026	(-0.01, 4E-03)	0.49	0.00085	(-6E-03, 6E-03)	0.77	-0.0014	(-6E-03, 3E-03)	0.53
AST	-0.0032	(-0.02, 0.02)	0.74	-0.000054	(-0.02, 0.03)	1	0.0012	(-0.01, 0.01)	0.85
Ferritin	0.00047	(5E-05, 9E-04)	0.03	0.000017	(-3E-04, 4E-04)	0.91	0.00011	(-9E-05, 3E-04)	0.28
Albumin	-0.21	(-1, 0.8)	0.68	0.31	(-0.8, 2)	0.6	-0.26	(-0.9, 0.4)	0.42
Hematrocrit	-0.13	(-0.4, 0.2)	0.39	1.1	(0.6, 2)	5E-05	0.24	(0.04, 0.4)	0.02
LDH	0.0026	(-3E-04, 6E-03)	0.08	0.0037	(-2E-03, 9E-03)	0.17	0.0025	(3E-04, 5E-03)	0.03
Hemoglobin	0.27	(-0.6, 1)	0.53	-2.7	(-4, -1)	2E-04	-0.61	(-1, -0.02)	0.04
CRP	-0.0011	(-7E-03, 4E-03)	0.71	0.0033	(-6E-03, 0.01)	0.49	0.0019	(-2E-03, 6E-03)	0.36
D-diimer	-0.00021	(-6E-04, 1E-04)	0.25	0.00039	(-1E-06, 9E-04)	0.07	0.000017	(-2E-04, 2E-04)	0.87
**Best model with biomarkers**									
	**In-sample**			**Out-of-sample**			**Total**		
	**β**	**β 95% CI**	***P***	**β**	**β 95% CI**	***P***	**β**	**β 95% CI**	***P***
Age	0.10	(0.06, 0.1)	1E-06	0.085	(0.04, 0.1)	0.002	0.089	(0.06, 0.1)	4E-09
BMI	0.027	(-0.03, 0.09)	0.38	0.018	(-0.06, 0.1)	0.65	0.024	(-0.02, 0.07)	0.29
HFrEF	2.3	(0.1, 4)	0.03	1.2	(-0.4, 3)	0.14	1.5	(0.3, 3)	0.01
Non-Hispanic White	0.85	(-0.4, 2)	0.22	-0.29	(-2, 2)	0.79	0.45	(-0.6, 2)	0.4
CKD	0.56	(-0.4, 1)	0.24	-0.44	(-2, 0.8)	0.49	0.33	(-0.3, 1)	0.34
ALC	-0.06	(-0.4, 0.05)	0.54	-0.10	(-1, 0.8)	0.84	-0.065	(-0.4, 0.05)	0.54
Lactate	0.24	(-0.07, 0.6)	0.14	-0.12	(-0.7, 0.4)	0.66	0.097	(-0.1, 0.3)	0.38
Procalcitonin	0.26	(0.08, 0.5)	0.01	0.085	(-0.07, 0.2)	0.24	0.15	(0.05, 0.3)	0.01
IL-1RA	1.4	(0.3, 3)	0.03	0.33	(-0.8, 2)	0.58	0.78	(0.07, 2)	0.05

CTSL1	0.46	(-0.4, 1)	0.27	0.50	(-0.4, 1)	0.27	0.49	(-0.06, 1)	0.09
ADAMTS13	-1.1	(-3, 0.4)	0.15	0.54	(-1, 2)	0.55	-0.11	(-1, 0.9)	0.83
VEGFD	-0.37	(-1, 0.3)	0.29	-0.53	(-2, 0.5)	0.32	-0.45	(-0.9, 0.04)	0.07
KIM1	-0.55	(-1, -0.1)	0.02	0.23	(-0.3, 0.7)	0.38	-0.20	(-0.5, 0.09)	0.19
ACE2	0.43	(-0.09, 1)	0.11	-0.13	(-0.9, 0.6)	0.71	0.17	(-0.2, 0.6)	0.39
IL6	0.37	(0.09, 0.7)	0.01	0.22	(-0.06, 0.5)	0.11	0.26	(0.07, 0.4)	0.01

The coefficients and *P* values were estimated using the raw hospital lab and biomarker measurements for the in-sample, out-of-sample, and total patient population. Confidence interval, CI; heart failure with reduced ejection fraction, HFrEF; absolute lymphocyte count, ALC; alanine transaminase, ALT; blood urea nitrogen, BUN; estimated glomerular filtration rate, eGFR; white blood cells, WBC; aspartate aminotransferase, AST; lactate dehydrogenase, LDH; body mass index, BMI; chronic kidney disease, CKD; C-reactive protein, CRP.
